# Beyond one-way determinism: San Frediano’s miracle and climate change in Central and Northern Italy in late antiquity

**DOI:** 10.1007/s10584-021-03043-x

**Published:** 2021-03-20

**Authors:** Giovanni Zanchetta, Monica Bini, Kevin Bloomfield, Adam Izdebski, Nicola Vivoli, Eleonora Regattieri, Ilaria Isola, Russell N. Drysdale, Petra Bajo, John C. Hellstrom, Robert Wiśniewski, Anthony E. Fallick, Stefano Natali, Marco Luppichini

**Affiliations:** 1 Dipartimento di Scienze della Terra, University of Pisa, Pisa, Italy; 2 Istituto di Geologia Ambientale e Geoingegneria, IGAG-CNR, Rome, Italy; 3 Centre for Climatic Change Impact CIRSEC University of Pisa, Pisa, Italy; 4 Istituto Nazionale di Geofisica e Vulcanologia, INGV Sez. Pisa, Pisa, Italy; 5 Department of History, Cornell University, Ithaca, USA; 6 “Palaeo-Science & History” Independent Research Group, Max Planck Institute for the Science of Human History, Jena, Germany; 7 Institute of History, Jagiellonian University in Krakow, Krakow, Poland; 8 Istituto di Geoscienze e Georisorse, IGG-CNR, Pisa, Italy; 9 School of Geography, The University of Melbourne, Melbourne, Australia; 10 Department of Mineral Resources, Croatian Geological Survey, 10000 Zagreb, Croatia; 11 School of Earth Sciences, The University of Melbourne, Melbourne, VIC 3010 Australia; 12 Faculty of History, University of Warsaw, Warsaw, Poland; 13 Scottish Universities Environmental Research Centre, SUERC, East Kilbride, UK; 14 Dipartimento di Scienze della Terra, University of Florence, Florence, Italy

**Keywords:** Precipitation, Roman Empire, Miracles, Social feedbacks, Cultural change

## Abstract

Integrating palaeoclimatological proxies and historical records, which is necessary to achieve a more complete understanding of climate impacts on past societies, is a challenging task, often leading to unsatisfactory and even contradictory conclusions. This has until recently been the case for Italy, the heart of the Roman Empire, during the transition between Antiquity and the Middle Ages. In this paper, we present new high-resolution speleothem data from the Apuan Alps (Central Italy). The data document a period of very wet conditions in the sixth c. AD, probably related to synoptic atmospheric conditions similar to a negative phase of the North Atlantic Oscillation. For this century, there also exist a significant number of historical records of extreme hydroclimatic events, previously discarded as anecdotal. We show that this varied evidence reflects the increased frequency of floods and extreme rainfall events in Central and Northern Italy at the time. Moreover, we also show that these unusual hydroclimatic conditions overlapped with the increased presence of “water miracles” in Italian hagiographical accounts and social imagination. The miracles, performed by local Church leaders, strengthened the already growing authority of holy bishops and monks in Italian society during the crucial centuries that followed the “Fall of the Roman Empire”. Thus, the combination of natural and historical data allows us to show the degree to which the impact of climate variability on historical societies is determined not by the nature of the climatic phenomena per se, but by the culture and the structure of the society that experienced it.

**Supplementary Information:**

The online version contains supplementary material available at 10.1007/s10584-021-03043-x.

## Introduction: confronting miracles

**Fig. 1:**
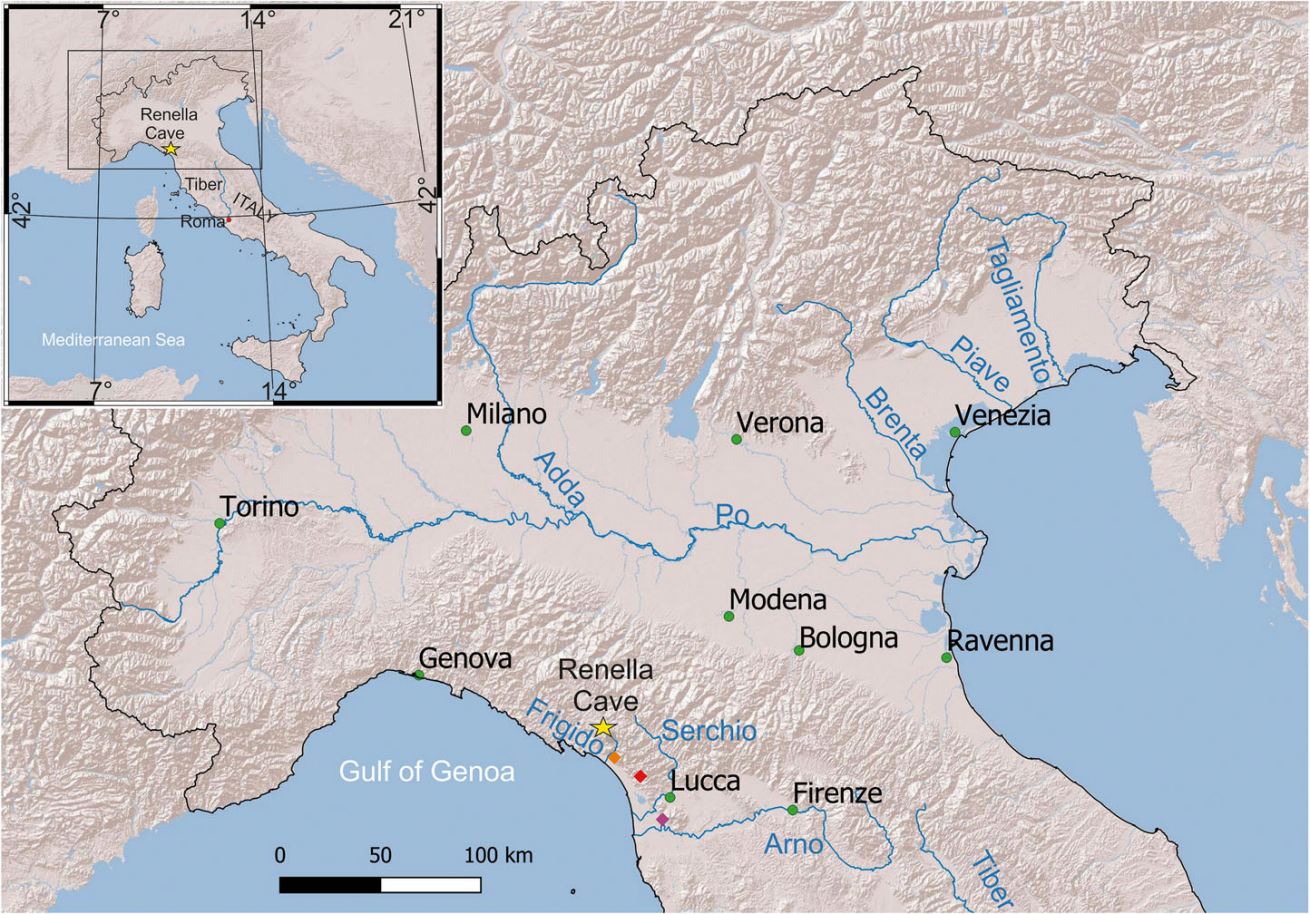
Location map of the places and rivers cited in the text. Orange diamond: Massa meteorological station; red diamond: S. Anna di Stazzema meteorological station; Purple diamond: Pisa meteorological station

Frediano (Latin: *Frigdianus*) was a bishop of the Italian town of Lucca in the sixth century AD (Central Italy, Fig. 1). Lucca was founded on the banks of the Serchio river, which at that time had several branches, and its unpredictable floods presented a continuous danger. The story of Frigdianus, told in the *Dialogues* (III 9), a hagiographical collection most probably written by pope Gregory the Great (late sixth c. AD) (de Vogüé 1978-1980) informs us that Frigdianus took a rake and made a track, and when the Serchio started to flood again, he lifted up his arms, ordering the river to follow the track. The river obeyed, changing its course and flowing farther from the town. One aim of the *Dialogues* was to propagate the cult of saints, so it is no surprise that the river listens to a holy bishop. What is surprising, however, is that this story is not an isolated case, but one element of a larger body of descriptions of hydroclimatic extremes and “water miracles” supposed to have taken place in Central and Northern Italy during the sixth c. AD (see Supplementary Tables S3).

Such a cluster of historical “climate records” has long been noticed. Since at least two centuries ago, many scholars have attempted to insert the Serchio River flooding within the framework of a general climatic deterioration (the “Medieval Deluge”) that supposedly occurred in late Roman and post-Roman Italy (fourth–seventh c. AD). Others tended to interpret the increasing frequency of floods to be a result of the progressive abandonment of the hydraulic systems of the Roman Empire, connected to the “barbarian invasions”—whereas at the same time, it may well be that we are dealing with a hagiographic *topos* (recurring literary motif) that should not be used as an independent basis for any palaeoclimatic reconstructions (for an overview of the debate, see e.g. Squatriti 1995).

Until now, all these lines of evidence either contradicted each other or resulted in a circular reasoning. Throughout the last decade, however, the palaeoclimate research on the Late Holocene achieved major progress, with new reconstructions appearing at a precipitous rate, and it is now possible to confront proxies derived from the natural archives with historical and archaeological data (cf. Izdebski et al. 2016a; Haldon et al. 2018b). An increasing number of records with high resolution indicate a complex pattern of climatic changes that affected Roman and late antique societies around the Mediterranean during the first millennium AD (e.g. Sadori et al. 2016). Paleoclimatologists identified a number of common trends of palaeoclimate variability across the Mediterranean, including the Late Roman Drier and Wetter Periods, or the Late Antique Little Ice Age (Izdebski et al. 2016a; Büntgen et al. 2016). Moreover, important attempts were made to link these climatic trends to major socio-economic transformations of the period (Izdebski et al. 2016a, Sadori et al. 2016, see also Sessa 2019 for an overview). As a result, in the context of the “Fall of the Roman Empire” or, more precisely, the disintegration of Roman hegemony in the western Mediterranean, a significant methodological debate is taking place on the consilience of natural scientific and historical-archaeological approaches to the study of the recent past (Sessa 2019; McCormick 2011; Izdebski et al. 2016b; Haldon et al. 2018a).

Crucially, the registration, nature and timing of these climatic changes, as well as their impact on the environment and society, are distressingly scarce for the very centre of the old Roman world: Italy. Here, we address this problem by providing a high-resolution oxygen isotope record with decadal resolution, i.e. a well-accepted paleohydrological indicator, for almost the entire first millennium AD (up to 900 AD), from a stalagmite from Renella cave in the Apuan Alps, not far from the city of Lucca (Fig. 1). In particular, we focus on the sixth c. AD, which turns out to be the wettest period in our record—and the sixth c. was more broadly a period of the strongest climatic change in the Mediterranean, when compared to the rest of the first millennium AD (Labuhn et al. 2018).

Central and Northern Italy indeed experienced a turbulent sixth c. AD. The region was the site of numerous battles and sieges during the decades-long Gothic War (535–554), as the eastern Romans under Justinian waged a campaign to reconquer the Italian peninsula from the barbarian Ostrogoths. In 568, only a few years following the end of the Gothic War, the Lombards crossed the Alps and invaded Northern Italy, turning the region into a battleground yet again. To the list of troubles may be added the arrival of the Plague of Justinian in Italy during the 540s, and which reappeared intermittently in the following decades, even if its death toll is far from being properly understood (Mordechai et al. 2019). By placing a rich dossier of textual data available for Central and Northern Italy from authors contemporary to the sixth c. AD, and in particular the numerous miracle accounts of the *Dialogues*, in the context of our palaeoclimatological study and the other published proxy data, we show how an increase in scientific knowledge can lead to a better understanding of the historical data. We demonstrate how this understanding reveals the dynamic interactions between climate, environment and society, which leads us to propose an approach to climate change studies that focuses on hybrid natural-cultural networks rather than unidirectional “deterministic” impacts.

### Site description

**Fig. 2:**
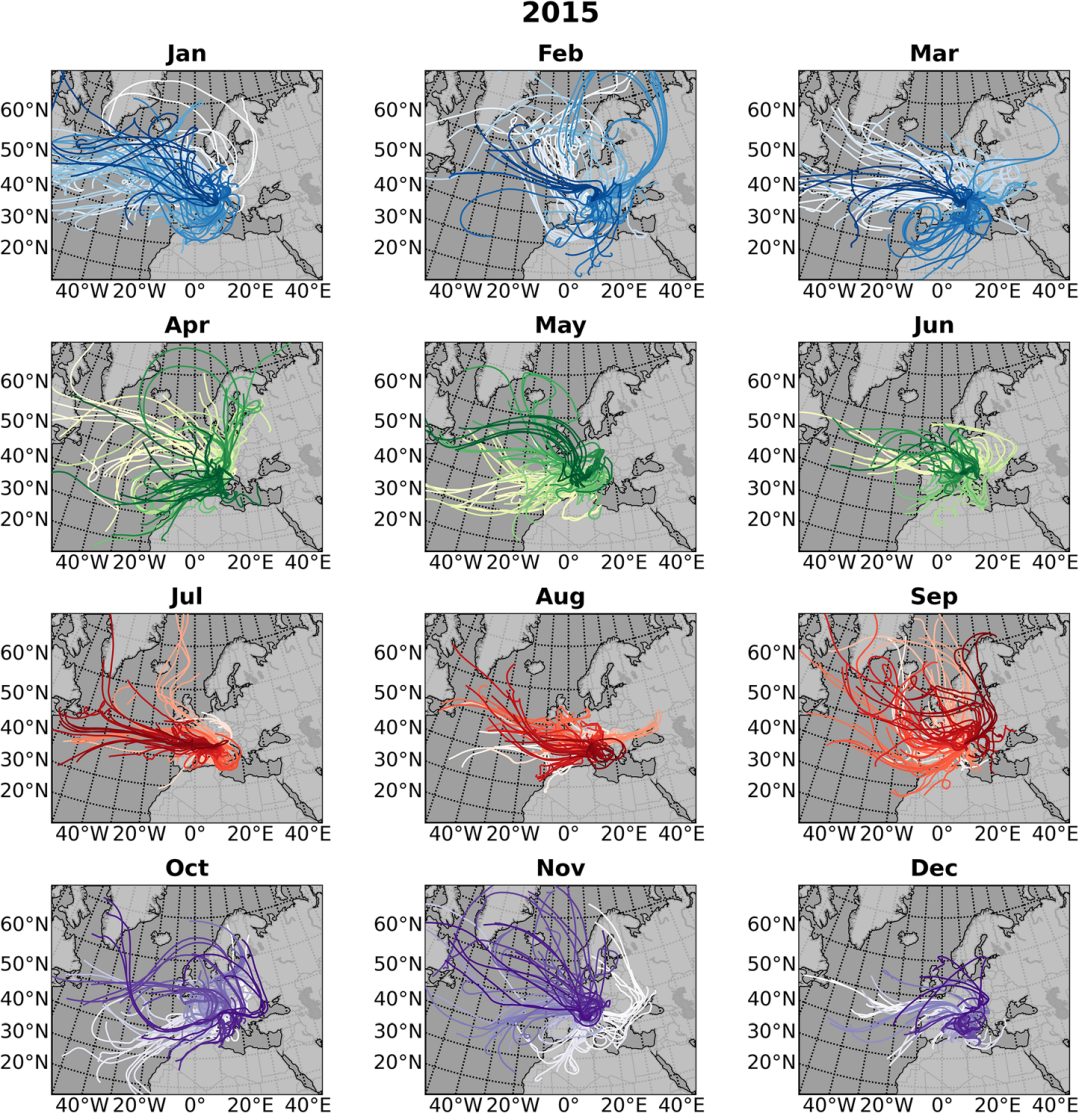
HYSPLIT back trajectory analyses for moisture arriving at Renella Cave during different months of the 2015. Meteorology from 1 × 1 degree Global Data Assimilation System 3-h meteorology reanalysis product (NCEP) (https://ready.arl.noaa.gov/HYSPLIT.php). For each month, we computed ⁓120 back trajectories (120 h) started four times daily at UTC 00, 06, 12, 18, and initialized at 1000 m (above ground level). Colours (blues, greens, reds, purples) indicate different seasons (winter, spring, summer, autumn), and the lightness value increases monotonically for each plot through the number of generated trajectories. The North Atlantic Oscillation (NAO) Index was positive in the 2015

Renella cave (44° 05′ 42″ N, 10° 11′ 01″ E, 275 m a.s.l) is a small, shallow cave opening onto the right slope of the Frigido River valley on the western side of the Apuan Alps (Figs. 1 and S1). The cave has a predominantly horizontal development within a Triassic metadolomite (Grezzoni Formation), close to the contact with the Palaeozoic phyllitic basement for a total length of ca. 200 m. The Apuan Alps experiences at low altitude a Mediterranean climate, but higher altitude mean annual precipitation can reach ca. 2000 mm (Piccini et al. 2008). Apuan Alps precipitation is predominantly of North Atlantic influence (López-Moreno et al. 2011; Luppichini et al. 2021). This is shown by HYSPLIT backward trajectory analyses for 2015 (Fig. 2), but a similar result can be found for other years. The air masses interact with the most important cyclogenesis centre of the Mediterranean region: the Gulf of Genoa (Trigo et al. 2002; Lionello et al. 2012). The local relief of the Apuan Alps and Apennines, which bound the eastern side of the Gulf of Genoa, plays an important role in trapping air masses moving eastward and triggering Genoa cyclones. The autumn/winter precipitation over the Apuan Alps and Northern Apennine are modulated by the North Atlantic Oscillation (NAO) synoptical pattern (López-Moreno et al. 2011, Luppichini et al. 2021, Fig. S2), with a negative (positive) index showing higher (lower) rainfall and the correlation is particularly pronounced during the winter months (Fig. S2, after Luppichini et al. 2021).

**Fig. 3:**
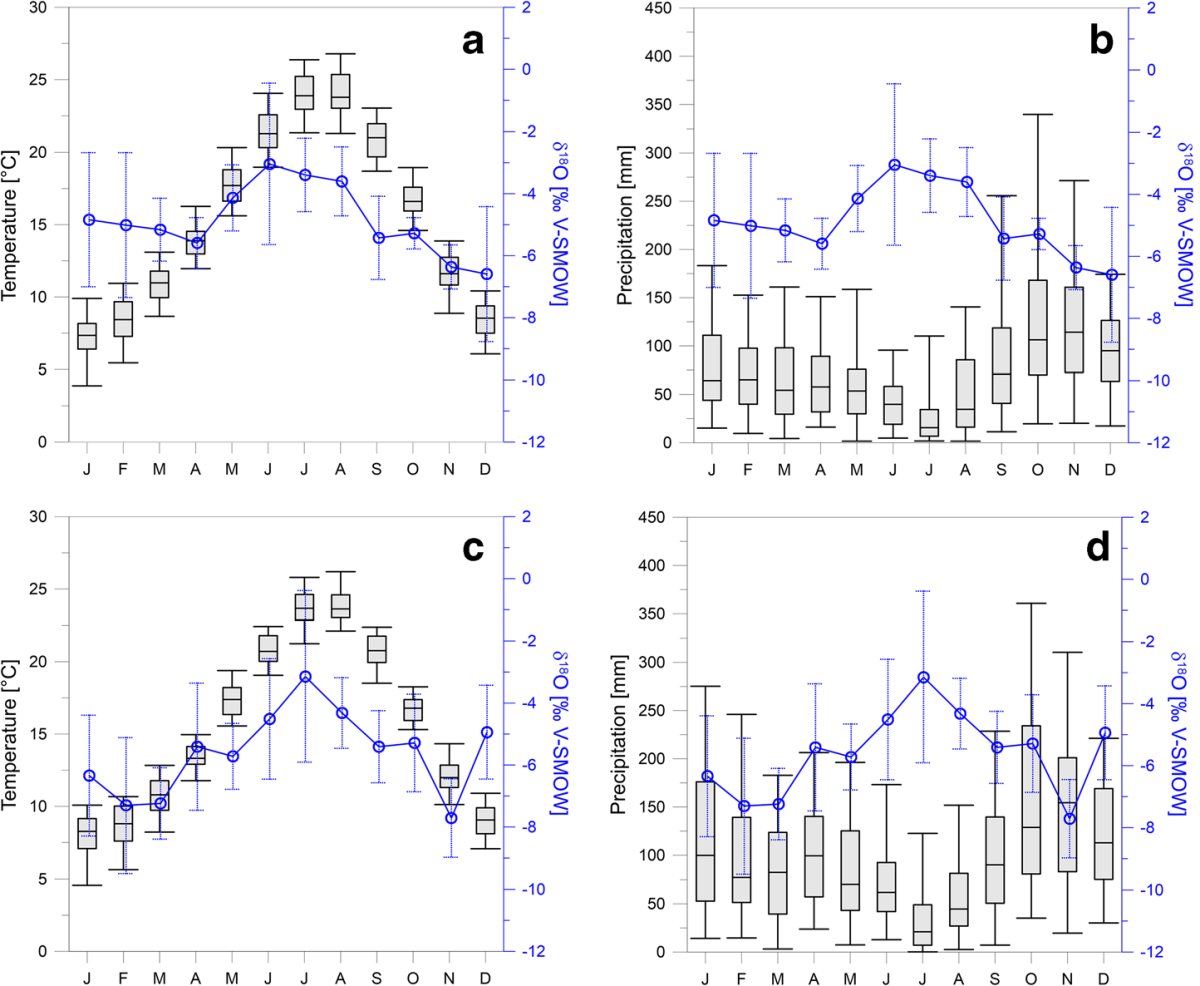
Modern climate in the area of Renella Cave. Box-and-whisker plots show monthly temperature and rainfall at meteorological stations of Pisa (A-B) and Massa (C-D) (https://www.sir.toscana.it/), 45 km south and 7 km southwest of Renella Cave, respectively. Daily time series of temperature and precipitation from 1942 to 2019 was used for Pisa, and from 1942 to 1999 for Massa. Lower and upper box boundaries indicate 25th and 75th percentiles, respectively, and line inside box marks the median. Whiskers above and below the box indicate the 5th and 95th percentiles. A, B) Monthly mean δ^18^O and standard deviation of precipitation (*n* = 6) at GNIP station in Pisa (IAEA/WMO 2019) along with meteorological data of Pisa. C, D) Monthly mean δ^18^O and standard deviation of precipitation (*n* = 5) at S. Anna di Stazzema (Doveri et al. 2019), 13 km southeast of Massa, over the box plot of Massa

The temperature for Renella cave is quite constant at ca. 13 °C, while mean monthly temperatures for the area range from 23.1 °C in July to 7.4 °C in January (Drysdale et al. 2006; Zhornyak et al. 2011 and Fig. 3). In addition to climatic data reported by Zhornyak et al. (2011), Fig. 3 shows the long-term temperature and precipitation data from the Pisa (Fig. 3A, B) and Massa (Fig. 3C, D) meteorological stations. These two stations are selected because of their proximity to two monitoring sites of stable isotopes in rainfall (Fig. 3 shows the δ^18^O data registered at Pisa (A, B) and S. Anna di Stazzema (C, D)). For the location of the stations, see Figs. 1 and S2.

**Fig. 4:**
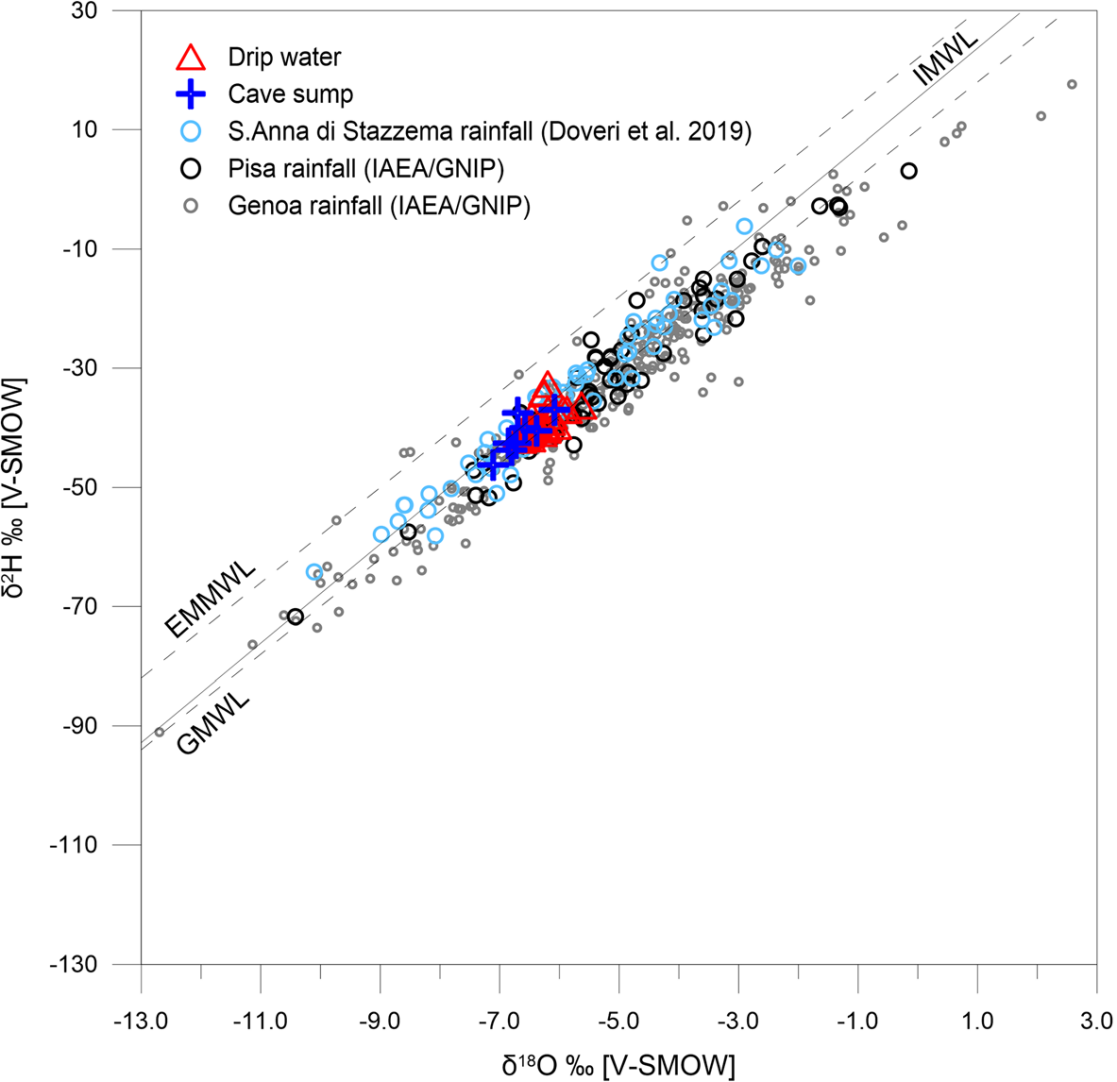
δ^2^H vs. δ^18^O diagram of Renella cave waters and local rainfall (Pisa and S. Anna di Stazzema). Both meteoric and cave waters overlap the Italian Meteoric Water Line (Giustini et al. 2016) between the GMWL (after Dansgaard 1964) and the meteoric water line of Eastern Mediterranean (EMMWL, Gat and Carmi 1970). Data of drip waters (*n* = 41) and sump waters (*n* = 10) come from Zanchetta et al. (2016) and two unpublished BSc and MSc thesis (Natali 2015; Tardelli 2016). Monthly δ^18^O of precipitation (*n* = 29) at GNIP station in Genoa (IAEA/WMO 2019) is also shown

Following the study of Drysdale et al. (2006), a discontinuous monitoring program was undertaken in the cave including cave water samples (drips, pools) for isotopic analyses (Zanchetta et al. 2016). However, the easy accessibility of the cave, with occasional visitors, produced several disturbances to the instrumentation, and long periods without significant dripping prevented accurate monitoring. Data gathering was also hampered by the presence of quarry activity, which may have disturbed the infiltration path. Data reported by Zanchetta et al. (2016) were improved with additional unpublished BSc and MSc theses (Natali 2015; Tardelli 2016). The isotopic composition of drips consistently lay on the local meteoric water line defined by Doveri et al. (2019), with an average δ^18^O composition of drips −6.23 ± 0.23‰ V-SMOW (Fig. 4), indicating that no evaporative effects are apparent in the infiltrated waters.

## Materials and methods

The sample for this study is a stalagmite (RL12) collected in the upper sector of the cave (Figs. S1 and 5), the most decorated chamber and where other samples were collected in the past (Drysdale et al. 2006; Zhornyak et al. 2011). The stalagmite was partially damaged on top, owing to the use of the cave for collection of speleothems for gardens in the past, and frequent visits of curious people and speleologists. The stalagmite was sectioned parallel to the growth axis, polished and sampled for stable isotopes, U/Th dating and thin sections.

Twelve samples in the form of small calcite prisms were extracted using a diamond drill for U/Th dating, which was performed at the University of Melbourne (Australia) following the method described by Hellstrom (2003). The samples were dissolved and a mixed ^236^U-^233^U-^229^Th spike was added prior to removal of the carbonate matrix with ion-exchange resin. The purified U and Th fraction diluted in nitric acid was introduced to a multi-collector inductively coupled plasma mass spectrometer (MC-ICPMS, Nu-Instruments Plasma). The ^230^Th/^238^U and ^234^U/^238^U activity ratios were calculated from the measured atomic ratios using an internally standardized parallel ion-counter procedure and calibrated against the HU-1 secular equilibrium standard. Correction for detrital Th content was applied using initial activity ratios of detrital thorium (^230^Th/^232^Th)_i_ of 1.5 ± 1.5 and further adjusted following the method described in Hellstrom (2006) using stratigraphic constraints. A depth-age model for RL12 was constructed using a Bayesian Monte Carlo approach (Scholz et al. 2012), following the method of Hellstrom (2006). Data are reported in Supplementary Table S1.

The samples for stable isotope ratios were obtained with an air-drill 0.8 mm drill bit for an average resolution of ca. 1.3 ± 0.3 mm. The stable isotope composition of the powders was measured using an AP2003 mass spectrometer at SUERC (East Kilbride, UK) on CO_2_ gas released by reaction with 105% H_3_PO_4_ at 70 °C. Isotopic results are reported using the conventional δ-notation in per mille (‰), with reference to the Vienna Pee Dee Belemnite (V-PDB) standard; the δ^18^O of waters cited in the text is quoted with reference to Vienna Standard Mean Ocean Water (V-SMOW). Mean analytical reproducibility (±1 σ) was ±0.06% and ± 0.07% for carbon and oxygen, respectively. Data are reported in Supplementary Table S2.

For this study, we searched through six major groups of textual sources (ancient and medieval texts in Greek or Latin) containing information on Central and Northern Italy in the period of 475–625 AD for records of hydrological extremes: (i) the works of a prolific early sixth c. writer Ennodius (Kennell 2000); (ii) the *Variae* (official letters) of Cassiodorus (the head of the civil government of Italy in the early sixth c.) (Bjornlie 2013); (iii) the *History of the Gothic Wars* by Procopius, an Eastern Roman historian (mid-sixth c.) (Kaldellis 2004); (iv) the letters and writings associated with pope Gregory the Great (late sixth c.) (Markus 1997); (v) mid-seventh c. brief biographies of Roman popes (Davis 2000); (vi) early-seventh c. historiographic traditions, no longer extant but preserved in a much later historiographic text (eighth c.) by Paul the Deacon (Capo 1992).

Water and fire miracles appearing in two late sixth-century collections of miracles have been counted: the *Dialogues* by Gregory the Great (appr. 133 miracle stories, set mostly in Italy) and the hagiographic corpus of Gregory of Tours (appr. 257 miracle stories, set mostly in Gaul), the latter served as a control group. This accounting is based on the records of the electronic Cult of Saints in Late Antiquity Database: http://csla.history.ox.ac.uk/. The records with water and fire miracles were identified using pre-defined database category “miracles over nature” and, additionally, searching for relevant terms in the sources. We have counted the ratio of water and fire miracles in relation to the total number of miracle stories in both collections. The statistical significance was tested using Fisher’s two-tailed exact test. For comparison, all other hagiographies in Greek, Latin, Syriac, Coptic Armenian and Georgian have been searched through using the CSLA database. We also compared our records of hydrological extremes from the Italian sources with a recent monograph on natural events in early medieval Europe (Wozniak 2020).

Moreover, in this article, we consider it to be of fundamental importance to connect the reconstruction of the variations of the hydrological regime (i.e. precipitation) offered by the oxygen isotopic composition of speleothems with the number of flood events obtained from textual sources, as well as the geoarchaeological and geological reconstruction. With this aim, a detailed critical review of the bibliography available for Central and Northern Italy was carried out from which the data for this work were selected.

## Results and discussion

### The palaeoclimatic reconstruction: the natural, historical and geoarchaeological data

The stalagmite shows some changes of growth axis, with the last phase developed on the side of the stalagmite. Within the stalagmite, there are at least 3 events of darker and sandier layers. In the upper part of the stalagmite, a gravel deposit is evident. This layer is composed of phyllite clasts, clear evidence of flooding of the cave from the nearby Canale Regolo (Zhornyak et al. 2011).

**Fig. 5:**
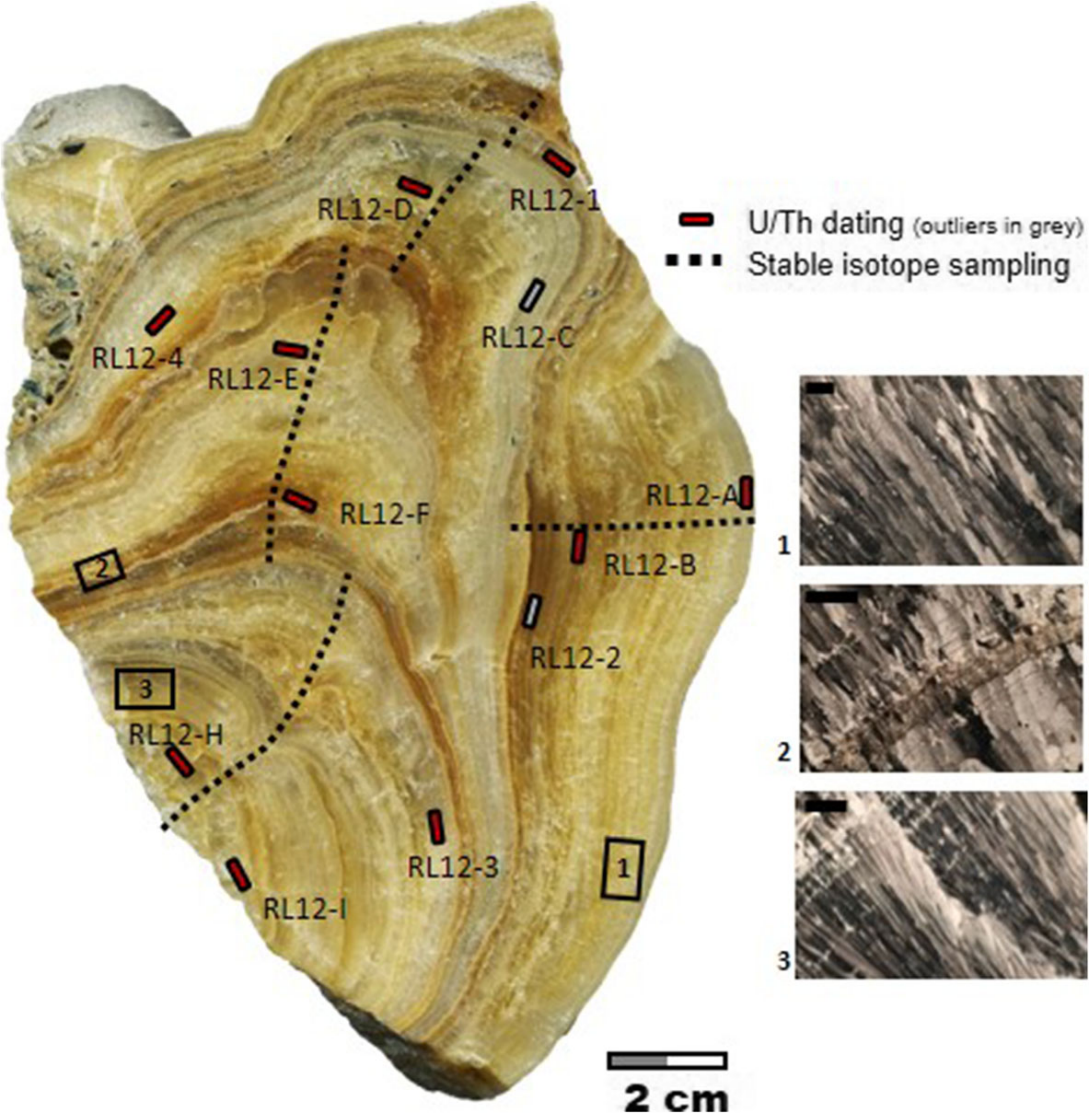
The figure shows the section of RL12 stalagmite with the traverse for the isotopic measurement and the location of samples for U/Th dating (for labelling, see Table S1). Selected petrographic features are also shown

The petrography shows that the stalagmite is mostly composed of compact columnar calcite with impurities, such as clastic material (Fig. 5) suggesting constant slow drip rate and near isotopic equilibrium condition of calcite precipitation (Frisia 2015).

**Fig. 6:**
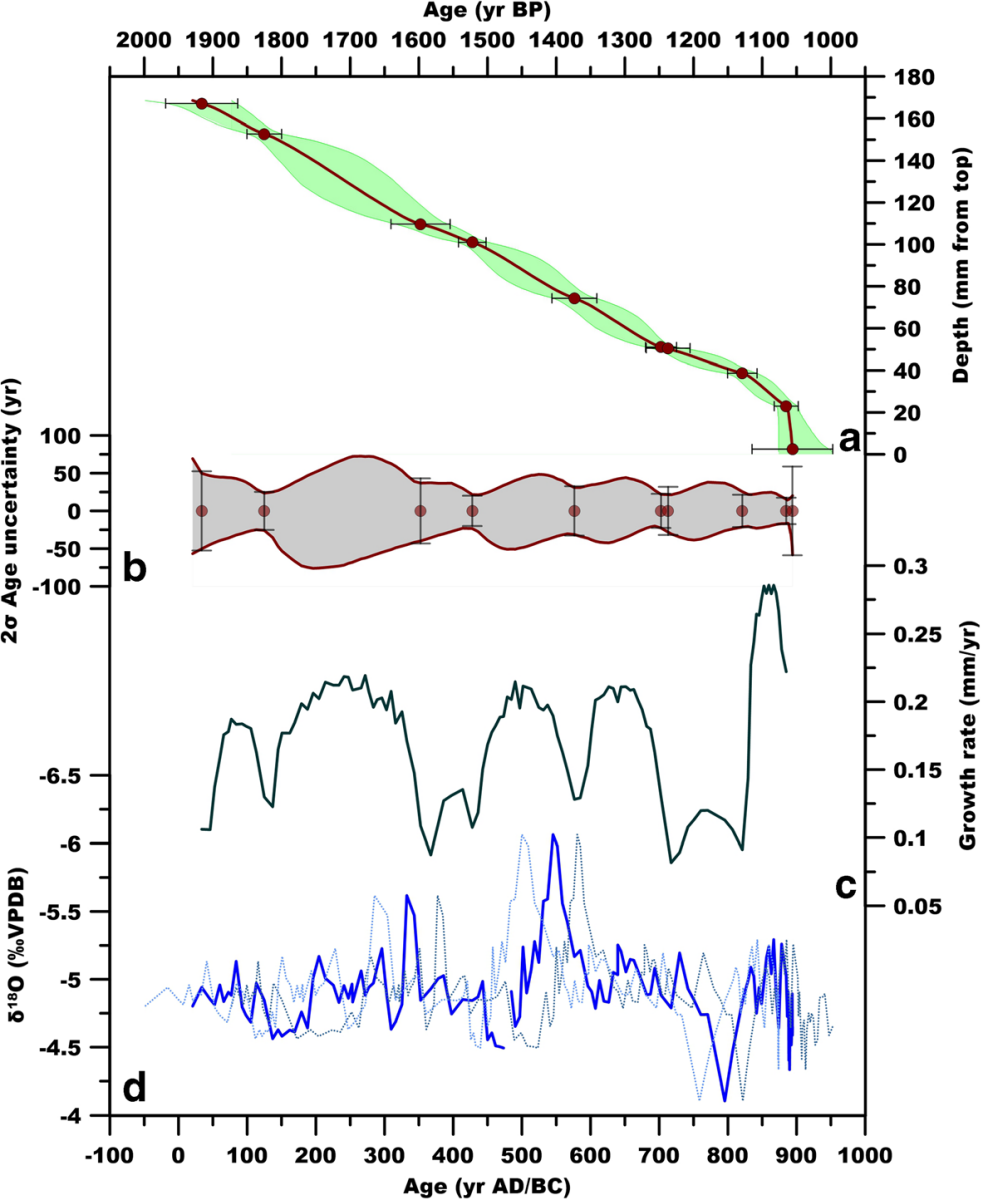
A) Age model for RL12 stalagmite (ages reported at 1950 AD). B) 2σ age uncertainty. C) Growth rate. D) RL12 δ^18^O time series (the thicker blue line represents the mean; the light blue lines represent ±2σ age uncertainty)

The age model is supported by 10 U/Th ages (two U/Th values were rejected as outliers), and reveals a growth rate ranging from 0.28 to 0.8 mm/year (Fig. 6) a mean stable isotope sample resolution of 7 ± 4 years and a mean age model error of 30 ± 14 years. The record spans between ca. 0 and 900 AD, and represents one of the most highly resolved and robustly dated records for this chronological interval in the central Mediterranean (e.g. Bini et al. 2020). The δ^18^O record shows several oscillations, with intervals of significantly above average values at ca. 140–170, 300–325, 450–495 and 750–820 AD. Intervals of lower δ^18^O values occur at ca. 330–345, 500–600, 630–675 and 845–885 AD, with the most prominent event represented by a century of lower δ^18^O values between ca. 500 and 600 AD. Considering age uncertainties, this multi-decadal period can be bracketed between ca. 450 and 650 AD (Fig. 6), centred in the sixth c.

The δ^18^O record in Mediterranean speleothems is mostly an indicator of hydrological conditions over the cave catchment and has been correlated to the amount of effective precipitation, with lower values indicating increasing rainfall (Drysdale et al. 2006; Bard et al. 2002; Bar-Matthews et al. 2003; Finné et al. 2017). This empirical observation is complex in nature and may be generated by different processes such as the seasonal distribution of precipitation (Longinelli et al. 2006), changes in air mass trajectories and the precipitation source (e.g. Celle-Jeanton et al. 2004); this is further complicated by processes such as recycled vapour (Dominguez-Villar et al. 2017). Baker et al. (2019) have recently shown that, in caves experiencing seasonal climate and mean annual temperature between >10 °C and < 16 °C, the drip water δ^18^O records the recharge-weighted δ^18^O of precipitation. In the case of Renella Cave, this signal should correspond mostly to the amount- weighted mean of autumn/winter precipitation. Investigation of precipitation δ^18^O time series shows that the IAEA station of Genoa has a negative correlation with NAO Index (Baldini et al. 2008), in agreement with the correlation between rainfall amount and NAO in the Central Mediterranean.

**Fig. 7:**
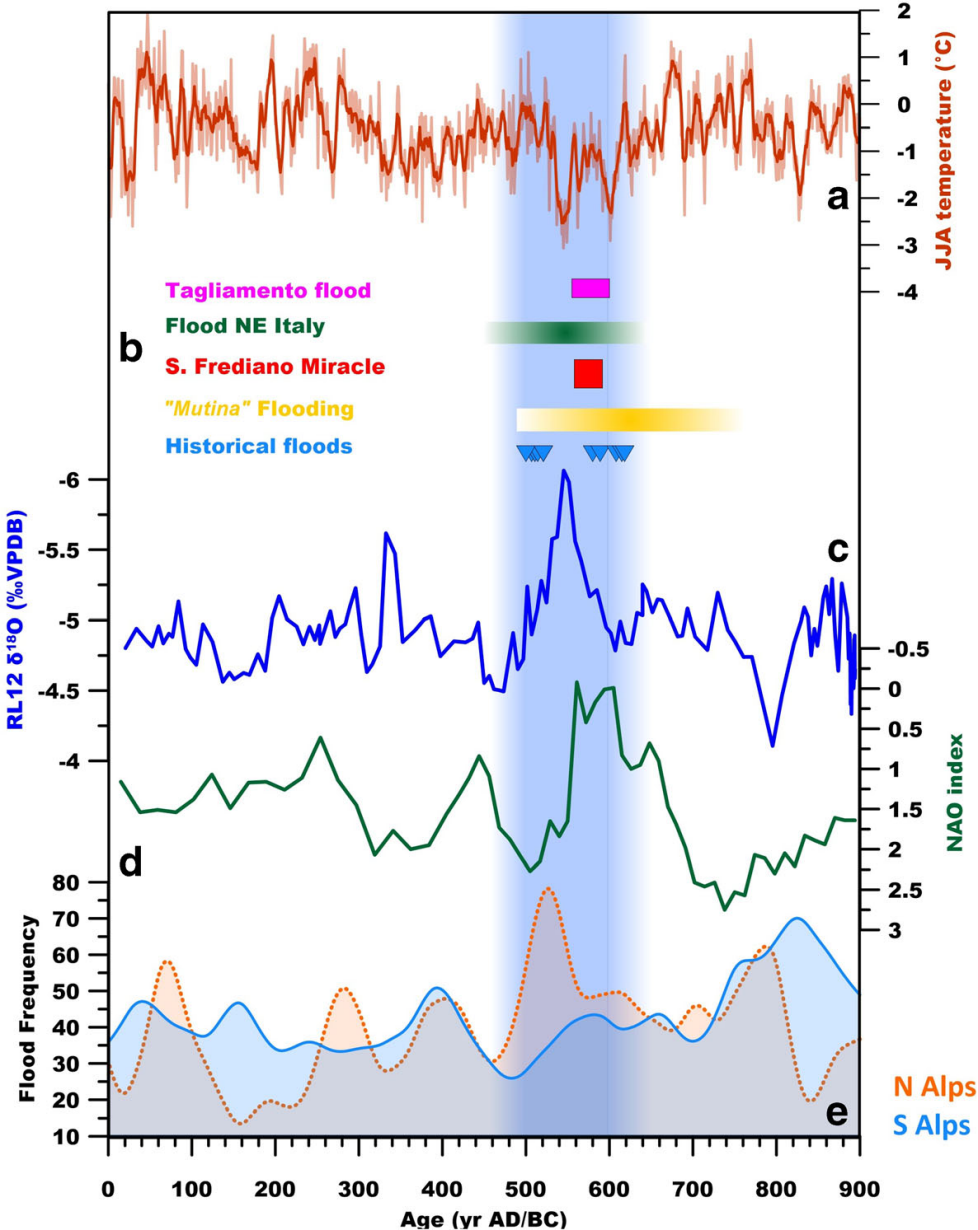
Comparison of the RL12 δ^18^O time series with different records. A) Reconstruction of summer temperature from dendroclimatological data from central Europe (Büntgen et al. 2016). B) Floods in central and northern Italy (Brenta, Piave and Tagliamento): NE Italy from Rossato et al. (2015); Tagliamento Flood from Fontana et al. (2020); Mutina flooding from Cremaschi and Gasperi (1989); historical floods from Supplementary Table S3 (this work). C) RL12 δ^18^O time series (this work). D) NAO index after Olsen et al. (2012). E) Flood frequency of central Alps (Wirth et al. 2013). Note that the chronology of historical floods based on radiocarbon dating can have a large associated error. The blue shaded box represents the interval of lower δ^18^O values of the RL12 time series including the ±2σ age uncertainty (from Fig. 6)

Therefore, the carbonate δ^18^O record indicates that regional climate during the period from the early Roman Empire to the early Middle Ages showed oscillations from drier to wetter conditions. However, as noted earlier the most profound feature of RL12 is the interval of lower δ^18^O values closely centred in the sixth c., which should correspond to an increase of autumn/winter cave recharge, favoured by an increase in moisture transport from the North Atlantic, which in turn may have enhanced secondary cyclogenesis in the Gulf of Genoa (Isola et al. 2019). These conditions are consistent, within age errors, with a more negative NAO index (Fig. 7) as reconstructed for this period by Olsen et al. (2012). Notably, this period of increased autumn/winter precipitation corresponds to a period of lower summer temperature in central Europe as deduced by dendroclimatological studies (Büntgen et al. 2016; Fig. 7) supporting the idea of generalised climatic changes in this period, with different regional expressions. In the case of the Renella record, its immediate spatial (regional) relevance is restricted to Central and Northern Italy, including the Po valley, based on the role of the Gulf of Genoa cyclogenesis for the rainfall in these parts of Italy.

Palaeoflood activity seems recurrent during the corresponding interval at local, regional and extra-regional scales. Geoarchaeological data indicate an increase of floods during the Late Antiquity in the Northern Apennine, albeit data are sparse (Bini et al. 2020). The most striking example is the alluvial sedimentation that suddenly started in the sixth c. and led to burial of the remains of the ancient city of Modena (Cremaschi and Gasperi (1989); the “Mutina” flooding, Fig. 7). This is not completely in agreement with the geoarchaeological data from nearby Bologna, where Cremonini et al. (2013) reported that riverbed aggradation became evident immediately after the third century AD. However, the palaeoflood record from NE Italian rivers (Brenta, Piave and Tagliamento, Rossato et al. 2015, Fig. 1) shows a consistent increase in the short interval between ca. 450 and 650 AD (Rossato et al. 2015), with prominent flood centred at 558–602 AD (Fontana et al. 2020) (Fig. 7). The data are consistent with increased flooding in river valleys of the central Alps obtained from different proxies (Wirth et al. 2013, Fig. 7).

A higher frequency of extreme rain events and floods is also suggested by the textual data available for Central and Northern Italy in the period of ca. 475–625 AD (Supplementary Table S3). Except for Procopius (group (iii), see Methods), all these texts provide descriptions of extreme hydroclimatic events occurring in Central and Northern Italy in the period of interest (Supplementary Table S3). Procopius is an exception that is not easy to explain. His lack of reference to the Italian hydroclimatic extremes may result from the fact that they did not have an obvious impact on the course of warfare, which was the main focus of his narrative, but this is hard to prove. Perhaps more importantly, unlike other authors whose works we searched through, Procopius was an outsider in Italy, who spent only a short time in the Peninsula (Cameron 1985). Not only his direct observations were limited but also even if he saw or was told about most abundant rains and floods, he could hardly realise they were more dramatic than those which the local population had witnessed in the past.

Therefore, historical records and the palaeoproxy data support the idea of an increase of rainfall and flood frequency closely centred in the sixth c. Nevertheless, the relation between flooding, climate and literature is not necessarily straightforward. Floods are produced by specific meteorological events characterised by a specific intensity and duration, and depend on soil moisture, catchment size and landscape conditions. Moreover, recognition of floods in the past in different archives can be challenging. Historical accounts do not record floods in a regular manner: for each source (historical text), this depends on a number of factors, such as the constraints of a specific literary genre, aims of the author, or the proximity to the period surveyed by the authors (cf. Sessa 2019; Squatriti 2010; Aldrete 2007). Altogether, this may lead to an underrepresentation of floods in the historical record as floods also likely occurred in places and times which are not documented in any texts. Similarly, geoarchaeological data may be incomplete and discontinuous (e.g. Bini et al. 2020). However, if floods or storms reflect the occurrence of extreme events, they can also be more frequent in a specific synoptic climatic condition. For instance, in some European regions, flood frequency has been related to the NAO (e.g. Villarini et al. 2012; Blöschl et al. 2019). According to Zanchettin et al. (2008), the wintertime river discharge peak of the Po river in the last ca. 200 years increases with the negative NAO index. In fact, a historical reconstruction of the damaging hydrological events in Peninsular Italy during the last 1000 years suggests that floods are tied to the NAO status, with increased frequency characterising the periods of negative NAO, for instance during the Little Ice Age (Diodato et al. 2019).

### Connecting the natural and the social: hydroclimatic extremes and Christian miracles

All these lines of evidence agree with the impression we get from the analysis of the historical textual information presented in Table S3. Without the palaeoclimatic proxies, this evidence should be discarded as anecdotal, reflecting a particular moment of interest in miracles produced by local saints (as argued by Squatriti 1995). With the new information on the climate of late antique Italy, gained from Renella cave, we can argue that these written sources plausibly reflect the actual environmental stress faced by Italians. As we already observed in the previous section, the hydroclimatic descriptions presented in Table S3 already constitute a significant number of records. What is more, they come from different types of texts, from almost every quarter century of Italian history in the sixth c., and can thus be treated as reflecting the mental-cultural frameworks used by subsequent generations of clerical writers to interpret the extreme hydroclimatic events in sixth-century Italy. In the course of the sixth c., in Italy’s literary culture, hagiography gained stronger ground compared to classical Latin literary genres and consequently mere reporting of hydroclimatic events changed into providing them with a super-natural meaning (see Tables 1 and S3). Admittedly, there is no way to say whether this is how the sixth-century lay peasants and city dwellers saw this experience; but this is certainly how the clerical writers wanted them to see it.

**Table 1:** Water miracles in the *Dialogues* of Gregory the Great (ed. Adalbert de Vogüé, Paris 1980)

#	Type	Reference	Saint	Location	Description
1	New source	II 5, 2–3	Benedictus	Campania	On a mountain.
2	New source	III 16, 2	Martinus	Campania	In a cave.
3	Torrential rain	II 33, 2–4	Scholastica	Campania	The saint woman brings about a torrential rain.
4	Torrential rain	III 11, 5	Cerbonius	Elba	The ship with the bishop’s body spared from a sudden rain.
5	Torrential rain	III 12, 3	Fulgentius	Otricoli	The bishop is saved from a torrential rain.
6	Torrential rain	III 15, 18	Euthicius	Norcia	The bishop’s relics are used to bring about a strong rain when it is too dry (repeatedly).
7	Flooding	III 9, 2–3	Frigdianus	Lucca	The bishop changes the course of the river Auser (Serchio).
8	Flooding	III 10, 2–3	Sabinus	Piacenza	The bishop orders the flood of the river Po to recede.
9	Flooding	III 19, 1–3	Zeno	Verona	The church of the saint saved from the flooding waters of the river Adige.

Crucially, the presence of the hydroclimatic events in the late sixth c. ecclesiastical (clergymen-produced) sources hints at the role that the increased precipitation and flooding could have played in the process of the socio-cultural change that was occurring at that time. After the devastating Gothic Wars in Italy in the middle of the sixth c., many earlier social institutions, based on urban civic communities and local secular elites were no longer functioning, and we have a number of indications that toward the end of the century bishops were becoming the leaders of the local communities (Izdebski 2012). At the same time, the cult of saints, unknown before Late Antiquity—and the belief in their power—was spreading through Italy. The main hagiographic sources from that time are the *Dialogues* of Gregory the Great, composed at the end of the sixth c. They contain numerous miracles (broadly falling into three types) that can be related to the hydroclimatic changes reconstructed by the RL12 δ^18^O record (Supplementary Table S2). Please note, however, that we do not interpret these miracles as records of hydroclimatic extremes taking place at the time when the saints described by Gregory had lived (potentially, several decades earlier), but rather as a testimony to Gregory’s special interest in this type of events. Admittedly, water miracles can be found already in the Bible (it suffices to mention Moses dividing the Red Sea and drawing water from the rock or Jesus walking on the lake) and so Gregory’s stories could theoretically be discarded as topical, reflecting rather pious readings of the author than real anxieties of his time. And it was a common *topos* in the hagiographical literature to depict saints as possessing power over the elements (Wozniak 2020, 53). However, on a closer examination, we realise that only some of Gregory’s water miracles have strong parallels in earlier Christian literature, while others are either new or strangely overrepresented in the *Dialogues*.

First, there are miracles related to sources of water (springs) appearing in new places (Table 1, # 1–2; Latin text and English translation of miracles from Table 1 is available in Table S4). These stories indeed have some literary precedents. However, such changes could actually be related to increased winter precipitation in this period (cf. Hirschfeld 2004 on springs in the Roman province of Palestine that are dry today, but were active during the period of increased precipitation in Late Antiquity; cf. also Izdebski et al. (2016b) on similar cases in the context of urban water supply in the same period).

Second, there are four miracles with various saints or their relics being able to invoke a torrential rain or a storm of unusual severity (Table 1, # 3–6). Such hydroclimatic extremes should have indeed been occurring with higher frequency in the sixth c. AD.

Third, there are several flood events in the *Dialogues* during which the saint miracle-makers show their power over the forces of nature (Table 1, # 7–9). Interestingly, already A. de Vogüé, the editor of the *Dialogues* (1978–80, vol. 2, p. 287–291; 347–349 [1]), saw in these miracles a reflection of real experience rather than hagiographical motifs, but he did not develop this intuition. Admittedly, one might argue that the sheer number of water miracles mirrors nothing more than the late flourish of Italian hagiography, which only in the sixth century produced a number of miracle stories. But the comparison of Gregory’s water miracles with a large body of late antique hagiographical literature shows that this is not the case.

The Oxford Cult of Saints in Late Antiquity Database (containing all known testimonies related to the cult of saints up to AD 700) attests only three flood-related miracles in the Christendom-wide corpus of Christian narrative prior to the *Dialogues* (Supplementary Table S5). Moreover, unlike in the *Dialogues*, which boast many such miracles spread across the entire text, these three miracles constitute single, isolated cases in larger narratives. Still, similar to the *Dialogues*, they were related to the local environmental context, which further confirms our conclusion that the *Dialogues* show a special interest in “water miracles”, reflecting the general cultural world of late sixth c. Italian audience of these stories. Let us also observe that the water miracles appear in almost 20% of the overall miracle accounts in the *Dialogues* (9 accounts out of 52, or 9 out of 133 total individual miracles—some accounts contain several miracles), while a similar contemporary hagiographic corpus from Gaul (modern France, for which the results from Renella Cave are not relevant), consisting of three collections of miracles written by Gregory of Tours, describes such miracles only incidentally (4 out of 257 miracle accounts—based on a search in the Oxford Cult of Saints in Late Antiquity Database, see Supplementary Table S6). Statistically, water miracles in Gregory the Great are significantly overrepresented (Fisher’s exact test: *p* value = 0.0133). This overrepresentation is even more remarkable if compared to the stories in which the power of a saint protects against fire. They are eight in the Gallic hagiography of Gregory of Tours, and only 2 in the Italian corpus of Gregory the Great (Supplementary Table S7): in this case, the difference is not statistically significant (*p* value = 0.5049).

To conclude, we wish to point out that the presence of these “hydroclimatic miracles” in the *Dialogues* is indicative of the increased frequency of real hydroclimatic events of a similar type in sixth c. Italy. The probability of such events and hydrological changes certainly increased in this period, as shown by the RL12 δ^18^O record, and apparently this increased frequency of unusual hydroclimatic events provided occasions for talking about water miracles performed by the holy men and women. If we accept that the telling of miracle stories—and in particular their recording in the *Dialogues*—were a means of increasing the authority of the saints, living or dead, the hydroclimatic trend recorded by the RL12 δ^18^O record would have facilitated this process. This trend meant that unusual hydroclimatic and hydrological events became much more frequent. As a result, these events and the associated environmental risks became part of the everyday Italian experience. Consequently, being able to manage these ferocious forces of nature—in the literary imagination of the readers—was an important reason to recognise a saint’s authority (just as today we recognise the authority of a state for its ability to provide health care and disease protection). At the same time, the increased frequency of such events offered more occasions for the claim of the performance of miracles by the saints.

## Conclusions: from climatic determinism to hybrid cultural-natural networks

**Fig. 8:**
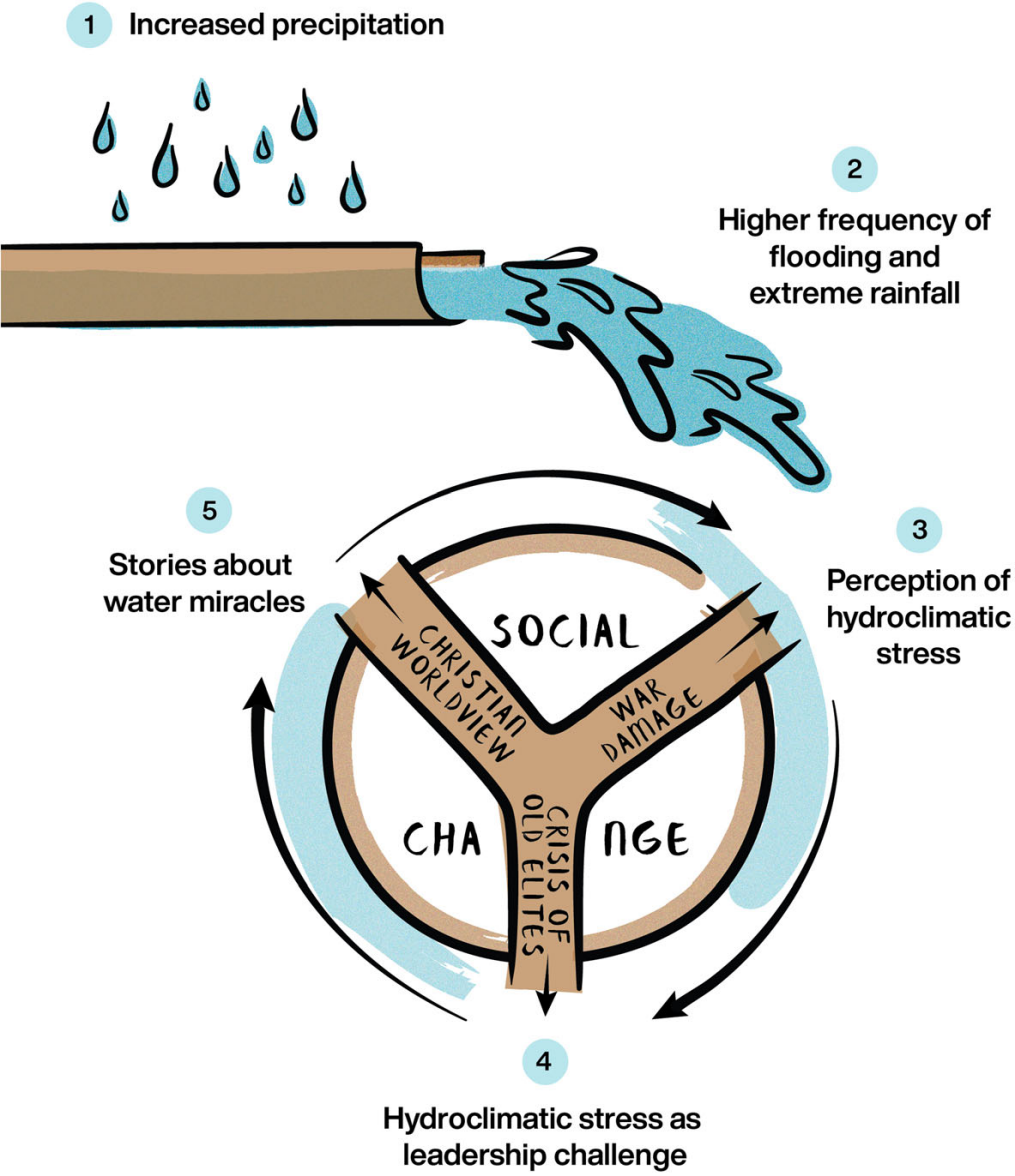
Schematic representation of the network of relationships between increased precipitation and social change in sixth c. Italy, as described in this article (drawing by Michelle O’Reilly, MPI SHH, design by Adam Izdebski)

Causing miracle stories to proliferate is not what one would expect as the key impact of a climatic change. In the traditional scheme of “climatic determinism”, subsistence crises and infrastructural damage would have been the main outcome, bringing about political or economic instability (Pfister and Brázdil 2006 for a more elaborate and balanced version of this approach). In other words, one expects a quasi-automatic reaction chain that is easy to predict: more rain causes floods, floods cause ruin—each climatic phenomenon should have its expected outcome. But nothing of that sort happened in sixth c. Italy. As we show in our synthetic Fig. 8 through a metaphor of the waterwheel, this was a more complicated and subtle process, in which a (nonhuman) climatic phenomenon assumed agency (i.e. had impact on the society) only through its integration into a complex network of (human) social actors (see Latour 2005 for the actor-network theory). The climatic phenomenon became part of the social dynamics and the cultural universe of a particular historical society; hence the scale and specificity of its impact depended on the society it “encountered”. Cultural and natural factors created together a single social-ecological system, a hybrid cultural-natural network.

To put it more generally, each society and culture has the potential to react differently to the same climatic change. For Cassiodorus, the most classicising (i.e. following the old Roman literary models) from among our sixth c. Italian writers, and a great administrator, floods were an opportunity for showing managerial prowess or lack thereof. In other historical contexts, Latin authors of classicising texts would present floods as signs of danger, for instance a flood of barbarians crossing the Alps (e.g. Ammianus 31.10; Seyfarth 1978), or on the contrary as cleansing waters protecting the land from civil war (Lucan, Pharsalia 4.115–20; Shackleton Bailey 1997). Post-classical chroniclers, such as Marius of Avenches (Table S3), would leave them without a comment. Our late sixth c. Italian churchmen and their audiences, instead, incorporated the hydroclimatic extremes into their social and cultural world as natural events with a super-natural dimension, open for divine intervention in the form of miracles—which in turn reinforced the belief in the power of God and his servants.

We are able to draw this conclusion because we can connect textual and natural scientific data. The δ^18^O record from the RL12 stalagmite we present here indicates a significant increase of precipitation in the sixth c. AD in Northern and Central Italy (please note this is a regionally specific effect—cf. Labuhn et al. 2018). It was potentially related to a more vigorous advection of vapour from the North Atlantic, stimulating secondary cyclogenesis in the Gulf of Genoa, in a general synoptical atmospheric circulation dominated by negative NAO conditions. As is evident from the geoarchaeological and historical textual evidence, this rise in precipitation resulted in increased frequency of floods in Central and Northern Italy.

The multi-disciplinary dataset we discuss in this paper also demonstrates that even though the critical analysis of historical textual sources often rightly leads to rejecting them as potential palaeoclimate proxies, when combined with the records derived from the natural archives and independent geoarchaeological investigation, their interpretation may change. In such multi-archive data-rich contexts, the textual data may be used for what they are best suited to: reconstructing cultural perceptions and societal dynamics related to the climatic and environmental change. Our case study thus shows how the situation of a seemingly “agonistic” consilience—that is of the different lines of evidence (textual, archaeological and environmental) leading to contradictory results (Sessa 2019)—can often be resolved by increasing data density. This could lead, as happened in our case, to both obtaining new evidence in favour of an existing hypothesis (increased flood frequency, though now precisely resolved in spatiotemporal terms) and gaining new insights (understanding the cultural reactions of the late Roman and post-Roman Italian society in the proper climatic context). Care taken to avoid falling into a climatic determinism in reading historical texts should not then devolve into a kind of social or cultural determinism that then denies any importance to climate for the analysis of these texts (as argued in Wozniak 2020, esp. p. 801). This new evidence-based “hybrid network” approach in the study of past climate impacts offers an opportunity to avoid simplistic, and hence catastrophist, interpretations, and brings our understanding closer to the real experience of past populations. At the same time, it also shows how different and unpredictable can be the reactions of our own societies to climatic changes in the near future.

## Supplementary information

ESM 1 (DOCX 11 kb)

ESM 2 (XLSX 16 kb)

ESM 3 (DOCX 33.6 kb)

ESM 4 (DOCX 1392 kb)

## Data Availability

New palaeoclimatic data used in this paper are available in Supplementary Table S2.
