# Supplementary material for: Beyond one-way determinism: San Frediano’s miracle and climate change in Central and Northern Italy in late antiquity
Source: Clim Change. 2021 Mar 20;165(1-2):25. doi: 10.1007/s10584-021-03043-x (PMC8550300; doi:10.1007/s10584-021-03043-x)
Supplement: Supplementary file 1 — (DOCX 11 kb) [file 10584_2021_3043_MOESM1_ESM.docx]

| Sample ID | Depth | ^238^U ng/g | ^230^Th/^238^U | ^234^U/^238^U | Age uncr (ka) | ^232^Th/^238^U | ^230^Th/^232^Th | **Age cr. (ka)** | *2σ (ka)* |
| --- | --- | --- | --- | --- | --- | --- | --- | --- | --- |
| RL12A-A | 2.5 | 624 | 0.0073 | 0.637 | 1.258 | 0.00538 | 1.4 | **1.122** | **0.059** |
| RL12A-B | 23.0 | 1243 | 0.0065 | 0.638 | 1.117 | 0.00010 | 63.2 | **1.123** | **0.018** |
| *RL12-2** | *28.5* | *1085* | *0.0069* | *0.620* | *1.221* | *0.00019* | *37.1* | ***1.212*** | ***0.031*** |
| RL12-1 | 38.7 | 1468 | 0.0061 | 0.563 | 1.189 | 0.00035 | 17.7 | **1.188** | **0.021** |
| *RL12A-C** | *41.2* | *1049* | *0.0117* | *0.604* | *2.137* | *0.00569* | *2.1* | ***1.976*** | ***0.067*** |
| RL12-4 | 50.5 | 1111 | 0.0072 | 0.606 | 1.305 | 0.00015 | 49.2 | **1.303** | **0.032** |
| RL12A-D | 51.0 | 1290 | 0.0077 | 0.641 | 1.318 | 0.00019 | 39.7 | **1.306** | **0.023** |
| RL12A-E | 74.3 | 1267 | 0.0082 | 0.624 | 1.444 | 0.00058 | 14.1 | **1.434** | **0.033** |
| RL12A-F | 101.0 | 1290 | 0.0090 | 0.624 | 1.587 | 0.00023 | 39.1 | **1.581** | **0.020** |
| RL12-3 | 109.7 | 1156 | 0.0093 | 0.609 | 1.680 | 0.00082 | 11.4 | **1.655** | **0.043** |
| RL12A-H | 152.7 | 1318 | 0.0111 | 0.636 | 1.923 | 0.00166 | 6.7 | **1.884** | **0.025** |
| RL12-I | 167.1 | 1341 | 0.0114 | 0.635 | 1.973 | 0.00013 | 89.6 | **1.975** | **0.053** |

Table S1-Corrected (in bold) and uncorrected U/Th ages for RL12 stalagmite. The activity ratios have been standardized to the HU-1 secular equilibrium standard, and ages calculated using decay constants of 9.195 ×10^−6^ (^230^Th) and 2.835 × 10^−6^ (^234^U). Depths are mm from top. Ages in grey (italic) marked with asterisk were rejected as outliers.
