# Supplementary material for: Beyond one-way determinism: San Frediano’s miracle and climate change in Central and Northern Italy in late antiquity
Source: Clim Change. 2021 Mar 20;165(1-2):25. doi: 10.1007/s10584-021-03043-x (PMC8550300; doi:10.1007/s10584-021-03043-x)
Supplement: Supplementary file 3 — (DOCX 33.6 kb) [file 10584_2021_3043_MOESM3_ESM.docx]

**Supplementary material – historical data**

Table S3. Mentions of hydroclimatic extremes said to have occurred in Central and Northern Italy in the period 475-625 CE.

| **#** | **Time** | **Type** | **Location** | **Reference** | **Comments** |
| --- | --- | --- | --- | --- | --- |
| 1 | 507-11 | Flood | Spoleto | Cassiodorus, *Variae* II 21 | Order to drain swamp in Umbria near Spoleto |
| 2 | 507-11 | Flood | Pontine Marshes | Cassiodorus, *Variae* II 32 | Proposal to dredge Pontine Marshes to stop the frequent flooding |
| 3 | 514-21 | Flood | Po valley | Ennodius, *Letters* 1.6 | Flood of Lake Como and the river Adda, a tributary of the Po |
| 4 | Unknown. Ca. 500-21 perhaps? | Flood | Po river | Ennodius, *Poems* 1.5 | Flood of the Po river. |
| 5 | Ca. 535 | Flood | Italy | Cassiodorus, *Variae* X 26 | Flooding of an unknown monastery from excessive rainfall |
| 6 | 536/7 | Drought | Northern Italy | Cassiodorus *Variae*, XII 25-27 | Drought in Liguria and Venetia (but not Istria, as specifically stated), affecting agricultural productivity. |
| 7 | 568 | Increased snowfall | Italy (North?) | Paul the Deacon, *History of the Lombards* II 10,14-16 | A lot of snow early on in the winter - and a bountiful harvest in the summer. Information potentially coming from a lost text of Secundus of Trent composed around the 620s. |
| 8 | 580 | Floods | S Switzerland, SW France (Rhone valley) and Italy (North?) | Marius of Avenches, *Chronicle*, anno 580 (p. 239); Gregory of Tours, *Histories* V 33 | Floods in October. A contemporary source. |
| 9 | 589 | Floods | Tiber, Adige, Northern Italy (?) | *The Book of Pontiffs,* p. 160; Gregory of Tours, *Histories*  X 1; Paul the Deacon, History of the Lombards III 23-24; Gregory the Great, *Dialogues* III 19,2-3; IV 18,2; IV 27,6; IV 37,7; IV 40,3 | Large floods on the Tiber, Adige (cf. the famous miracle in Verona) and potentially also other N Italian rivers. The contemporary sources (Gregory the Great, Gregory of Tours) focus on the situation in Rome and Verona. Paul the Deacon, writing two centuries later but perhaps using contemporary sources which are now lost,  mentions that the floods occurred also in other Venetian and Ligurian cities. |
| 10 | 591 | Drought (?) | Central Italy (Latium?) | Gregory the Great, *Register of Letters* I 70; Paul the Deacon, *History of the Lombards* IV 2 | Pope Gregory the Great, in his (contemporary) letter mentions bad harvests in the region of Rome (but no hydroclimatic event), without giving reasons. Paul the Deacon, writing some two centuries later, reports a severe drought lasting from January to September of 591. The pope's letter does not describe the situation as dramatic, it is rather usual business of making provisions in a situation of increased risk of grain shortages in Rome in the early spring. Hence, this probably was not a major decrease in the winter season precipitation. |
| 11 | 608/615 | Floods | Rome | *The Book of Pontiffs,* p. 165 | This is one sentence mentioning “famine, plague, and flood”, without a precise date. It comes from a text written some 30-40 years later (cf. Davis 2000). There is no way evaluating if these were regular inundations of the Tiber, or a major flood: the context focuses on overall hardship, probably related to the period of civil war and chaos in the Eastern Roman Empire, on which the papacy depended. Given the time distance between the event and the writing of this text, the floods might have been a vague association, a later conjecture inspired by the events of the famous floods of AD 589-590. |
| 12 | 618 | Flood (?) | Tiber | Paul the Deacon, *History of the Lombards* IV 45 | The text suggests this flood was related to an earthquake, so probably cannot be used as evidence of a hydroclimatic extreme. |

Table S4. Latin text and English translation of water miracles in the *Dialogues* of Gregory the Great (ed. Adalbert de Vogüé, Paris 1980; trans. Gardner 1911).

| # | Type | Reference | Text (de Vogüé) | Translation (1911 translation) |
| --- | --- | --- | --- | --- |
| 1 | New source | II 5,2-3 | Quos blande consolatus dimisit, et nocte eadem cum paruo puerulo nomine Placido, cuius superius memoriam feci, eiusdem montis ascendit rupem, ibi que diutius orauit, et oratione conpleta, tres petras in loco eodem pro signo posuit, atque ad suum, cunctis illic nescientibus, monasterium rediit.  Cumque die alio ad eum pro necessitate aquae praedicti fratres redissent, dixit: "ite, et rupem illam, in qua tres super inuicem positas petras inuenitis, in modico cauate.  Valet enim omnipotens deus etiam in illo montis cacumine aquam producere, ut uobis laborem tanti itineris dignetur auferre".  Qui euntes rupem montis, quam Benedictus praedixerat, iam sudantem inuenerunt, cumque in ea concauum locum fecissent, statim aqua repletus est, quae tam sufficienter emanauit, ut nunc usque ubertim defluat atque ab illo montis cacumine usque ad inferiora deriuetur. | The man of God, comforting them with sweet words, caused them to return back again; and the next night, having with him only the little boy Placidus (of whom we spake before), he ascended up to the rock of that mountain, and continued there a long time in prayer; and when he had done, he took three stones, and laid them in the same place for a mark, and so, none of them being privy to that he had done, he returned back to his own Abbey. And the next day, when the foresaid monks came again about their former business, he said thus unto them: "Go your way to the rock, and in the place where you find three stones laid one upon another, dig a little hole, for almighty God is able to bring forth water in the top of that mountain, and so to ease you of that great labour which you take in fetching it so far." Away they went, and came to the rock of the mountain according to his direction, which they found as it were sweating drops of water, and after they had with a spade made an hollow place, it was straightways filled, and water flowed out so abundantly, that it doth plentifully, even to this day, spring out and run down from the top to the very bottom of that hill. |
| 2 | New source | III 16,2 | Cuius hoc miraculum primum fuit, quod, mox se in praedicti montis foramine contulit, ex petra eadem, quae in semetipsa concaua angustum specum fecerat, aquae stilla prorupit, quae Martino dei famulo in usu cotidiano sufficeret, et nec plus adesset nec necessitati deesset. Qua in re ostendit omnipotens deus quantam sui famuli curam gereret, cui uetusto miraculo potum in solitudine ex petrae duritia ministraret. | His first miracle was that, so soon as he made choice of that cave for his habitation, there sprung water out of the hollow rock, which was neither more nor less than served for his necessity: by which almighty God did shew what great care he had of his servant, seeing miraculously, as in ancient time he had before done to the children of Israel, he caused the hard rock to yield forth water. |
| 3 | Torrential rain | II 33,2-4 | Tanta uero erat caeli serenitas, ut nulla in aere nubes appareret. Sanctimonialis autem femina, cum uerba fratris negantis audisset, insertas digitis manus super mensam posuit, et caput in manibus omnipotentem dominum rogatura declinauit. Cumque leuaret de mensa caput, tanta coruscationis et tonitrui uirtus tanta que inundatio pluuiae erupit, ut neque uenerabilis benedictus, neque fratres qui cum eo aderant, extra loci limen quo consederant pedem mouere potuissent. Sanctimonialis quippe femina, caput in manibus declinans, lacrimarum fluuios in mensam fuderat, per quos serenitatem aeris ad pluuiam traxit. Nec paulo tardius post orationem inundatio illa secuta est, sed tanta fuit conuenientia orationis et inundationis, ut de mensa caput iam cum tonitruo leuaret, quatenus unum idem que esset momentum et leuare caput et pluuiam deponere. | At that time, the sky was so clear that no cloud was to be seen. The Nun, receiving this denial of her brother, joining her hands together, laid them upon the table: and so, bowing down her head upon them, she made her prayers to almighty God: and lifting her head from the table, there fell suddenly such a tempest of lightning and thundering, and such abundance of rain, that neither venerable Benedict, nor his monks that were with him, could put their head out of door: for the holy Nun, resting her head upon her hands, poured forth such a flood of tears upon the table, that she drew the clear air to a watery sky, so that after the end of her devotions, that storm of rain followed: and her prayer and the rain did so meet together, that as she lifted up her head from the table, the thunder began, so that in one and the very same instant, she lifted up her head and brought down the rain. |
| 4 | Torrential rain | III 11,5 | Defuncti igitur corpus inposuerunt naui. Cumque Populonium tenderent, collecto in nubibus aere, inmensa nimis pluuia erupit. Sed ut cunctis patesceret, cuius uiri corpus nauis illa portaret, per illud maris spatium quod ab Helba insula usque Populonium duodecim millibus distat, circa utraque nauis latera procellosa ualde pluuia descendit, et in naui eadem una pluuiae gutta non cecidit. | For performing of this his will they provided a ship, and away they went with his body towards Populonium: in which journey there fell great store of rain, but that the world might know whose body was transported in that ship, in that twelve miles' space which is betwixt the island and Populonium, a great storm of rain fell upon both sides of the ship, but not one drop within. |
| 5 | Torrential rain | III 12,3 | Quem dum feroces Gothi, ministri scilicet crudelitatis illius, tenuissent, circumdantes eum, uno in loco stare praeceperunt, ei que in terra circulum designauerunt, extra quem pedem tendere nullo modo auderet". Cumque uir dei in sole nimio staret, ab eisdem Gothis circumdatus et designatione circuli inclausus, repente coruscus et tonitruus et tanta uis pluuiae erupit, ut hii, qui eum custodiendum acceperant, inmensitatem pluuiae ferre non possent. Et dum magna nimis inundatio fieret, intra eandem designationem circuli, in qua uir dei fulgentius stetit, ne una quidem pluuiae gutta descendit. | The merciless Goths executed his cruel commandment: and setting him upon a piece of ground, they made a circle round about him, out of which they commanded him not to stir his foot. Whiles the man of God stood there in great extremity of heat, environed round about with those Goths, suddenly there fell such thunder and lightning, and such plenty of rain, that his keepers could not endure that terrible storm: and yet for all that, not one drop fell within the circle, where the man of God, Fulgentius, stood. |
| 6 | Torrential rain | III 15,18 | Euthicius uero, qui praedicti Florentii in uia dei socius fuerat, magis post mortem claruit in uirtute signorum. Nam cum multa ciues urbis illius de eo soleant narrare miracula, illud tamen est praecipuum, quod usque ad haec Langobardorum tempora omnipotens deus per uestimentum illius assidue dignabatur operari. Nam quotiens pluuia deerat et aestu nimio terram longa siccitas exurebat, collecti in unum ciues urbis illius eius tunicam leuare atque in conspectu domini cum precibus offerre consueuerant. Cum qua dum per agros exorantes pergerent, repente pluuia tribuebatur, quae plene terram satiare potuisset. Ex qua re patuit eius anima quid uirtutis intus, quid meriti haberet, cuius foris ostensa uestis iram conditoris auerteret. | But Euthicius, who was companion to Florentius in serving of God, was famous also for miracles after his death. For the inhabitants of that city do speak of many: but the principal is that which, even to these times of the Lombards, almighty God hath vouchsafed to work by his coat: for when they had any great drought the citizens, gathering themselves together, did carry that, and together with their prayers offer it in the sight of our Lord. And when they went with that through the fields, praying to God, forthwith they had such plenty of rain as the dryness of the ground required: whereby it was apparent, what virtue and merits were in his soul, whose garment shewed outwardly did pacify the anger of almighty God. |
| 7 | Flooding | III 9,2-3 | …quod Ausarit fluuius, qui iuxta urbis illius muros influebat, saepe inundatione facta cursus sui alueum egressus, per agros diffundi consueuerat, et quaeque sata ac plantata repperiret euerteret. Cumque hoc crebro fieret et magna eiusdem loci incolas necessitas urgueret, dato studio operis, eum per loca alia deriuare conati sunt. Sed quamuis diutius laboratum fuisset, a proprio alueo deflecti non potuit. Tunc uir domini Frigdianus rastrum sibi paruulum fecit, ad alueum fluminis accessit et solus orationi incubuit, atque eidem flumini praecipiens ut sequeretur, per loca quaeque ei uisa sunt rastrum per terram traxit. Quem, relicto alueo proprio, tota fluminis aqua secuta est, ita ut funditus locum consueti cursus desereret, et ibi sibi alueum, ubi tracto per terram rastro uir domini signum fecerat, uindicaret, et quaeque essent alimentis hominum profutura sata uel plantata ultra non laederet. | Hard by the walls of the city, there runneth a river called Auser, which divers times doth so swell and overflow the banks, that it drowneth many acres of ground, and spoileth much corn and fruit. The inhabitants, enforced by necessity, seeing that this did often happen, went about by all means possible to turn the stream another way: but when they had bestowed much labour, yet could they not cause it to leave the old channel. Whereupon the man of God, Frigidianus, made a little rake, and came to the river, where all alone he bestowed some time in prayer; and then he commanded the river to follow him, and going before, he drew his rake over such places as he thought good, and the whole river, forsaking the old channel, did follow him, and kept possession of that which the holy man by that sign of his rake had appointed: and so never afterward did it hurt any more either corn or other things planted for the maintenance of men. |
| 8 | Flooding | III 10,2-3 | In ea namque ciuitate Sabinum nomine fuisse asserunt mirae uirtutis episcopum. Cui dum die quadam suus diaconus nuntiasset, quod cursus sui Padus alueum egressus ecclesiae agros occupasset totaque illic loca nutriendis hominibus profutura aqua eiusdem fluminis teneret, uenerabilis uitae Sabinus episcopus respondit, dicens: "uade, et dic ei: mandauit tibi episcopus, ut te conpescas et ad proprium alueum redeas". Quod diaconus audiens despexit et inrisit. Tunc uir domini, arcessito notario, dictauit dicens: "Sabinus domini Iesu Christi seruus commonitorium Pado. Praecipio tibi in nomine Iesu Christi domini, ut de alueo tuo in locis istis ulterius non exeas, nec terras ecclesiae laedere praesumas". Atque eidem notario subiunxit dicens: "uade, hoc scribe, et in aquam eiusdem fluminis proice". Quo facto, sancti uiri praeceptum suscipiens, statim se a terris ecclesiae fluminis aqua conpescuit, atque ad proprium alueum reuersa, exire ulterius in loca eadem non praesumpsit. Qua in re, Petre, quid aliud quam inoboedientium hominum duritia confunditur, quando in uirtute iesu et elementum inrationabile sancti uiri praeceptis oboediuit? | For in that town of Placentia, they say that there was a Bishop of wonderful virtue, called Sabinus: who understanding by one of his Deacons, that the great river of Po was broken forth, and had overflowed the land which belonged to the church, and done much harm, he bad him go unto the river, and deliver it this message from him: "The Bishop commandeth you to retire, and keep yourself within your own bounds." His Deacon, hearing these words, scornfully contemned to be employed in any such business. Then the man of God, Sabinus, sent for a notary, and willed him to write these words: "Sabinus, the servant of our Lord Jesus Christ, sendeth admonition to Po. I command thee, in the name of our Lord Jesus Christ, that thou come not out of thy channel, nor presume any more to hurt the lands of the church." This short letter he bad the notary write, and when he had so done, to go and cast it into the river. The notary did as he bad him, and the river obeyed the precept of the holy man, for straightways it withdrew itself from the church-lands, returned to his own channel, and never presumed any more to overflow those grounds. By which fact, Peter, the pride of disobedient men is confounded, seeing that the very senseless element, in the name of Jesus, obeyed the holy man's commandment |
| 9 | Flooding | III 19,1-3 | Huic tam antiquo miraculo diebus nostris res similis e contrario euenit elemento. Nam nuper Iohannes tribunus relatione sua me docuit, quod Pronuulfus comes, cum ilico adesset, se cum rege Authari eo tempore in loco eodem ubi mira res contigit adfuisse, eam que se cognouisse testatus est. Praedictus etenim tribunus narrauit dicens, quia ante hoc fere quinquennium, quando apud hanc Romanam urbem alueum suum Tiberis egressus est, tantum que crescens ut eius unda super muros urbis influeret atque in ea maximas regiones occuparet, apud Veronensem urbem fluuius atesis excrescens ad beati Zenonis martyris atque pontificis ecclesiam uenit. Cuius ecclesiae dum essent ianuae apertae, aqua in eam minime intrauit. Quae paulisper crescens, usque ad fenestras ecclesiae quae erant tectis proximae peruenit, sic que stans aqua ecclesiae ianuam clausit, ac si illud elementum liquidum in soliditatem parietis fuisset inmutatum. Cumque essent multi interius inuenti, sed, aquarum magnitudine ecclesia omni circumdata, qua possent egredi non haberent, ibique se siti ac fame deficere formidarent, ad ecclesiae ianuam ueniebant, ad bibendum hauriebant aquam, quae, ut praedixi, usque ad fenestras excreuerat et tamen intra ecclesiam nullo modo defluebat. Hauriri itaque ut aqua poterat, sed defluere ut aqua non poterat. Stans autem ante ianuam ad ostendendum cunctis meritum martyris, et aqua erat ad adiutorium et quasi aqua non erat ad inuadendum locum. Quod ego antiquo antedicti ignis miraculo uere praedixi non fuisse dissimile, qui trium puerorum et uestimenta non contigit et uincula incendit. | Like unto this ancient miracle we had in our days another, but yet in a divers element: for not long since John the Tribune told me that, when the Earl Pronulphus was there, and himself also with Antharicus the king, how there happened at that time a strange miracle, and he affirmeth that himself doth know it to be true. For he said that, almost five years since, when the river of Tiber became so great that it ran over the walls of Rome, and overflowed many countries: at the same time in the city of Verona, the river Athesis did so swell, that it came to the very church of the holy martyr and Bishop Zeno; and though the church doors were open, yet did it not enter in. At last it grew so high, that it came to the church windows, not far from the very roof itself, and the water standing in that manner, did close up the entrance into the church, yet without running in: as though that thin and liquid element had been turned into a sound wall. And it fell so out, that many at that time were surprised in the church, who not finding any way how to escape out, and fearing lest they might perish for want of meat and drink, at length they came to the church door, and took of the water to quench their thirst, which, as I said, came up to the windows, and yet entered not in; and so for their necessity they took water, which yet, according to the nature of water, ran not in: and in that manner it stood there before the door, being water to them for their comfort, and yet not water to invade the place: and all this to declare the great merit of Christ's martyr. Which miracle I said truly, that it was not unlike to that ancient one of the fire: which burnt the three children's bands, and yet touched not their garments. |

Table S5. Flood miracles in hagiographical sources prior to the *Dialogues* of Gregory the Great – from the entire Mediterranean, identified using the Cult of Saints in Late Antiquity Database - <http://csla.history.ox.ac.uk/>.

| # | Database ID | Reference | Saint | Approximate miracle time | Source composition date | Location | Description | Comments |
| --- | --- | --- | --- | --- | --- | --- | --- | --- |
| 1 | E00144 | *Stories of the Monks of the Desert* (Coptic) 53b-54a | Apa Aaron | 4^th^/10^th^ c. CE | 4^th^ c. CE (?) | Philae, Upper Egypt | When the yearly flood of the Nile had not risen to reach all the fields, those affected by the drought came to Apa Aaron and asked for help. He prayed and cried and thus God had compassion to raise the Nile water to flow over all the land. | The miracle is set in the environmental context of Egypt, whose agriculture depended on the annual Nile flood, which varied interannually, and could even be supressed by tropical volcanic eruptions (Manning et al. 2017). However, it is striking that this is the only flood-related miracle from Egypt, with its unique dependence on the Nile flooding, which shows how unusual is the prevalence of water miracles in the *Dialogues*. Please note, however, that the *History of the Monks of Egypt* tells about John of Lycopolis who predicted the flood of the Nile – no miracle, however, is involved. |
| 2 | E01878 | Gregory of Nyssa, *Life of Gregory the Miracle-Worker* 56-61 | Gregory the Miracle-Worker | 3^rd^ c. CE | 380s CE | Central Turkey | Gregory is invited by the inhabitants of a region affected by the floods of the river Lycus to help. He plants his staff in the mud of the river and prays. The staff becomes a tree and furnishes an unsurpassable boundary to the stream. His miracles are compared to those of Elijah. | Again, we are dealing with an isolated miracle, clearly inspired by a biblical *topos*. Its occurrence in a hagiographic text composed in Central Anatolia of the later 4^th^ c. CE may not be without significance, however: at that time, Central Anatolia experienced decline in winter precipitation (Izdebski et al. 2016), which could further aggravate the damaging impact of sudden floods caused by infrequent high rainfall – or, *a contrario*, the dry context could have increased the author’s sensibility to and interest in extreme hydrological events. |
| 3 | E02347 | Eugippius, *Life of Severinus* 15 | Severinus | Severinus died in 482 CE | Early 6^th^ c. CE | Noricum (upper Danube) | By cutting a cross on one of the posts supporting a wooden church at Quintanis, Severinus saves it from further flooding of the Danube. | In premodern times, floods of the Danube were frequent and they feature in ancient literature in different contexts (Stanfill and Schneider, 2017), so it is not surprising that they became part of the only late antique life of a saint who lived near the Danube. However, please note that this life was actually composed by a writer active in Italy in the early 6^th^ c. CE, so he could have already been experiencing the increased precipitation regime revealed in the Renella data published in this article. |

Table S6. Water miracles in the works of Gregory of Tours, a contemporary of Gregory the Great – based on the Cult of Saints in Late Antiquity Database - <http://csla.history.ox.ac.uk/>.

| # | Oxford CSLA ID | Gregory’s text | Description |
| --- | --- | --- | --- |
| 1 | E00627 | *Glory of the Martyrs* 83 | Gregory of Tours tells how his father acquired, in 533, relics of unnamed saints and was protected by them; how his mother repelled fire with them; and how he himself was saved from a storm by them. |
| 2 | E02635 | *Glory of the Confessors* 45 | A saint from his grave at Blaye (near Bordeaux) protected people travelling on the river Garonne from shipwreck; Gregory himself witnessed the calming of a storm and flood. |
| 3 | E05466 | *Life of the Fathers* 17,5 | Nectius “calmed a storm on a river with the sign of the cross.” |
| 4 | E07841 | *Glory of the Martyrs* 43 | A massive rainstorm fell in Clermont and flooded the rivers except a small area where relics were kept. |

Table S7. Fire miracles in the works of Gregory of Tours and in the *Dialogues* of Gregory the Great – based on the Cult of Saints in Late Antiquity Database - <http://csla.history.ox.ac.uk/>.

| **Fire miracles in Gregory of Tours’ hagiographical corpus (8)** |
| --- |
| E00039 Gregory of Tours writes the Life of *Gallus (bishop of Clermont, ob. 551, [S00034](http://csla.history.ox.ac.uk/record.php?recid=S00034)): it presents the saint as a man of exceptional patience, who protects Clermont (central Gaul) from fire, earthquake and plague; many miracles occur at his funeral and thereafter. From Gregory's Life of the Fathers, written in Latin in Tours (north-west Gaul), 573/594. Overview of Gregory's Life of Gallus. |
| E00383: In his Glory of the Martyrs, written in Latin, Gregory of Tours tells how relics of *Mary (Mother of Christ, [S00033](http://csla.history.ox.ac.uk/record.php?recid=S00033)), *Martin (ascetic and bishop of Tours, ob. 397, [S00050](http://csla.history.ox.ac.uk/record.php?recid=S00050)) and unnamed *Apostles ([S00084](http://csla.history.ox.ac.uk/record.php?recid=S00084)), which he wore on him in a golden cross, miraculously extinguished a fire consuming a poor man's house. Written in Tours (north-west Gaul), 580/594. |
| E00627: In his Glory of the Martyrs, written in Latin, Gregory of Tours tells how his father acquired, in 533, relics of unnamed saints and was protected by them; how his mother repelled fire with them; and how he himself was saved from a storm by them, and was subsequently taught a lesson in humility. Written in Tours (north-west Gaul), 580/594. |
| E02453: In his Glory of the Confessors, written in Latin, Gregory of Tours recounts how *Eusebius (bishop of Vercelli, ob. 371, [S01219](http://csla.history.ox.ac.uk/record.php?recid=S01219)), from his grave in Vercelli (northern Italy) cures people and casts out demons, particularly on his feast day, when the possessed smash the lamps and are cured by the falling oil. Gregory's mother placed relics of Eusebius in the oratory of her house in Gaul, which saved her and the building from fire. Written in Tours (north-west Gaul), 587/588. |
| E02646: In his Glory of the Confessors, written in Latin, Gregory of Tours tells how the cloth covering the tomb of *Melanius (bishop of Rennes, ob. c. 530, [S01279](http://csla.history.ox.ac.uk/record.php?recid=S01279)) in Rennes (north-west Gaul) remained undamaged through a great fire. Written in Tours (north-west Gaul), 587/588. |
| E02650: In his Glory of the Confessors, written in Latin, Gregory of Tours recounts how *Victorius (bishop of Le Mans, ob. c. 490, [S01280](http://csla.history.ox.ac.uk/record.php?recid=S01280)) saved the city of Le Mans (north-west Gaul) from fire; people are cured at his tomb. Written in Tours (north-west Gaul), 587/588. |
| E04488: In his Miracles of Martin, written in Latin, Gregory of Tours describes how the bishop's residence in Poitiers (western Gaul) was protected from fire by dust from the tomb of *Martin (ascetic and bishop of Tours, ob. 397, [S00050](http://csla.history.ox.ac.uk/record.php?recid=S00050)) at Tours, which was kept by the bishop of Poitiers in a holy container; AD 591. Written in Tours (north-west Gaul), 591/594. |
| E04638: In his Miracles of Martin, written in Latin, Gregory of Tours recounts how prayers to *Martin (ascetic and bishop of Tours, ob. 397, [S00050](http://csla.history.ox.ac.uk/record.php?recid=S00050)) prevented fire from spreading in Bordeaux (south-west Gaul); and how one of his servants was healed of dysentery after swallowing dust from Martin's tomb in Tours; AD 593/594. Written in Tours (north-west Gaul), 593/594. |
| **Fire miracles in the *Dialogues* (2)** |
| E04432: Gregory the Great, writing in Latin in c.593 in Rome, describes how *Marcellinus (a 6th c. bishop of Ancona, [S01712](http://csla.history.ox.ac.uk/record.php?recid=S01712)) miraculously caused a fire to change its course in the city of Ancona, northern Italy (*Dialogues* I 6). |
| E04482: Gregory the Great, writing in Latin in c. 593 in Rome, describes how *Benedict (6th c. hermit near Rome, [S01759](http://csla.history.ox.ac.uk/record.php?recid=S01759)), remained unharmed as Goths attempted to burn him alive in his hut, near Rome (*Dialogues* III 18) |

**Bibliography for historical data**

**Critical editions of historical sources referenced in Tables S3 and S4**

Gregory the Great, Dialogues, ed. A. de Vogüé, Sources Chrétiennes 252, 260, and 265, Paris: Cerf, 1978-1980.

Book of Pontiffs (Liber Pontificalis) anonymous, ed. Th. Mommsen, Monumenta Germaniae Historica, Gesta Pontificum Romanorum, Berlin: Weidmann, 1898.

Cassiodorus, Variae ed. Th. Mommsen, Monumenta Germaniae Historica, Auctores Antiquissimi 12, Berlin: Weidmann, 1894.

Ennodius, Opera. ed. F. Vogel, Monumenta Germaniae Historica, Auctores Antiquissimi 7, Berlin: Weidmann, 1885.

Gregory of Tours, Histories, ed. B. Krusch and W. Levison, Monumenta Germaniae Historica, Scriptores rerum Merovingicarum 1, Hannover: Hahn, 1884.

Gregory the Great, Register of Letters, ed. P. Norberg, Corpus Christianorum. Series Latina 140-140A, Turnhout: Brepols, 1982.

Marius of Avenches, Chronicle, ed. Th. Mommsen, Monumenta Germaniae Historica, Auctores Antiquissimi 11, Berlin: Weidmann, 1894.

Paul the Deacon, History of the Lombards, ed. L. Bethmann and G. Waitz, Monumenta Germaniae Historica, Scriptores rerum Langobardicarum, Hannover: Hahn, 1978.

**Other bibliography**

Gardner, E., trans. The dialogues of Saint Gregory, surnamed the Great; pope of Rome & the first of that name. London: P. L. Warner, 1911

A. Izdebski, J. Pickett, N. Roberts, T. Waliszewski, The environmental, archaeological and historical evidence for regional climatic changes and their societal impacts in the Eastern Mediterranean in Late Antiquity. *Quaternary Science Reviews* **136**, 189–208 (2016).

J. G. Manning, *et al.*, Volcanic suppression of Nile summer flooding triggers revolt and constrains interstate conflict in ancient Egypt. *Nature Communications* **8**, 900 (2017).

J. P. Stanfill, A. W. Schneider, Gothia Submerged: The Impacts of Severe Flooding on Valens’s First Gothic War. *Journal of Late Antiquity* **10**, 351–371 (2017).
