# Supplementary material for: Beyond one-way determinism: San Frediano’s miracle and climate change in Central and Northern Italy in late antiquity
Source: Clim Change. 2021 Mar 20;165(1-2):25. doi: 10.1007/s10584-021-03043-x (PMC8550300; doi:10.1007/s10584-021-03043-x)
Supplement: Supplementary file 4 — (DOCX 1392 kb) [file 10584_2021_3043_MOESM4_ESM.docx]

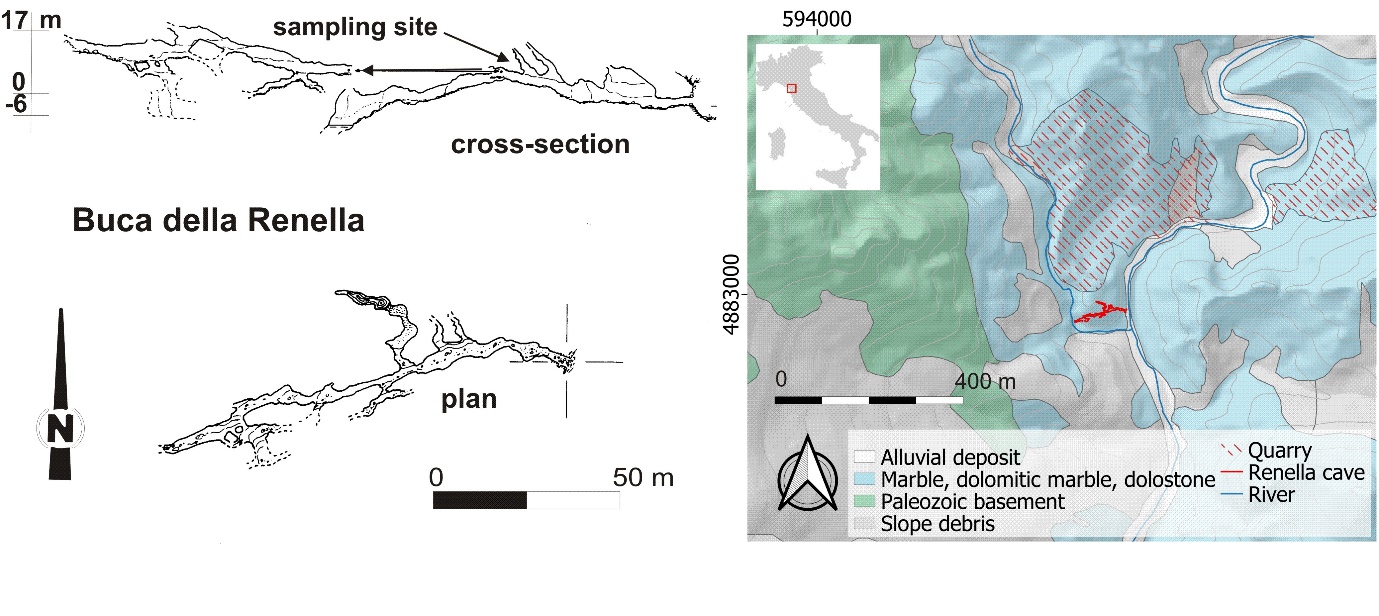


Figure S1 - Cave geology and plan view and cross section and sampling site (after Zhorniak et al., 2011).


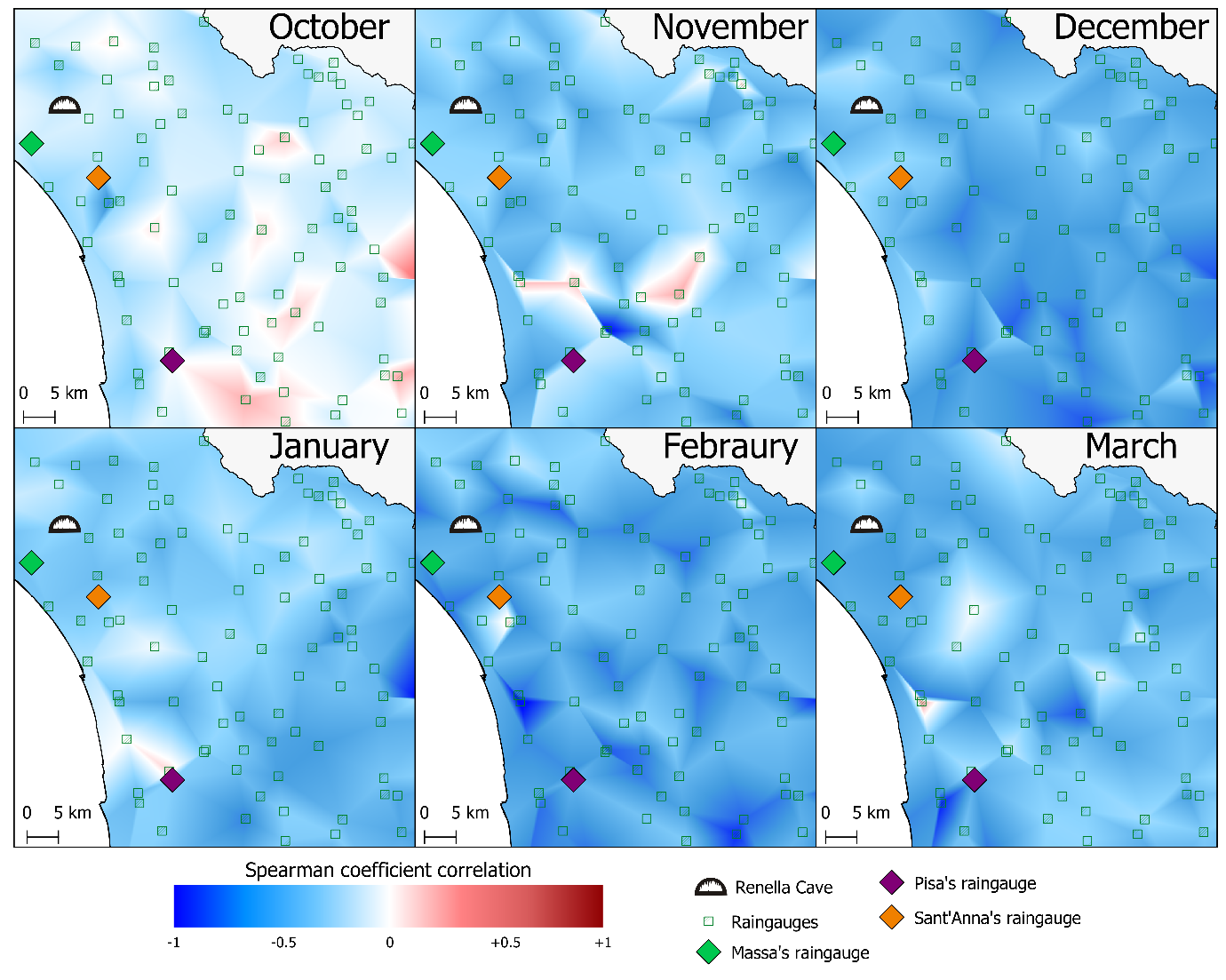


Figure S2 - Spatial distribution of Spearman’s coefficient of correlation (SCC) between North Atlantic Oscillation (NAO) Index and winter monthly rainfall in northern-western Tuscany. The processing was carried out using 91 stations of the regional network of the Regional Hydrological Service (green squares). The NAO dataset used is provided by the Climate Analysis Section of the US National Center for Atmospheric Research (NCAR). This dataset is based on the difference of normalized sea level pressure (SLP) between Lisbon, Portugal and Stykkisholmur/Reykjavik, Iceland since 1864 (<https://climatedataguide.ucar.edu/climate-data/hurrell-north-atlantic-oscillation-nao-index-station-based>). Data analyses are after Luppichini et al., submitted. The processing was obtained using raingauges with a period of activity > 30 years and using rainfall and NAO dataset between 1920 and 2019. The raingauges of the regional network of Pisa (purple rhombus) and Massa (green rhombus) and the raingauge of Sant'Anna (orange rhombus) are shown.
